# Development of a clinical metagenomics workflow for the diagnosis of wound infections

**DOI:** 10.1186/s12920-024-02044-w

**Published:** 2024-11-25

**Authors:** Carl Halford, Thanh Le Viet, Katie Edge, Paul Russell, Nathan Moore, Fiona Trim, Lluis Moragues-Solanas, Roman Lukaszewski, Simon A. Weller, Matthew Gilmour

**Affiliations:** 1 Defence Science and Technology Laboratory, Porton Down, Salisbury, Wiltshire, UK; 2 University of East Anglia, Norwich, Norfolk, UK; 3 Quadram Institute Bioscience, Norwich Research Park, Norwich, Norfolk, UK; 4 Salisbury NHS Foundation Trust, Salisbury Hospital, Salisbury, UK; 5 Hampshire Hospitals NHS Foundation Trust, Basingstoke and North Hampshire Hospital, Basingstoke, UK

**Keywords:** Molecular diagnostics, Clinical metagenomics, Wound infections, Nanopore sequencing

## Abstract

**Background:**

Wound infections are a common complication of injuries negatively impacting the patient’s recovery, causing tissue damage, delaying wound healing, and possibly leading to the spread of the infection beyond the wound site. The current gold-standard diagnostic methods based on microbiological testing are not optimal for use in austere medical treatment facilities due to the need for large equipment and the turnaround time. Clinical metagenomics (CMg) has the potential to provide an alternative to current diagnostic tests enabling rapid, untargeted identification of the causative pathogen and the provision of additional clinically relevant information using equipment with a reduced logistical and operative burden.

**Methods:**

This study presents the development and demonstration of a CMg workflow for wound swab samples. This workflow was applied to samples prospectively collected from patients with a suspected wound infection and the results were compared to routine microbiology and real-time quantitative polymerase chain reaction (qPCR).

**Results:**

Wound swab samples were prepared for nanopore-based DNA sequencing in approximately 4 h and achieved sensitivity and specificity values of 83.82% and 66.64% respectively, when compared to routine microbiology testing and species-specific qPCR. CMg also enabled the provision of additional information including the identification of fungal species, anaerobic bacteria, antimicrobial resistance (AMR) genes and microbial species diversity.

**Conclusions:**

This study demonstrates that CMg has the potential to provide an alternative diagnostic method for wound infections suitable for use in austere medical treatment facilities. Future optimisation should focus on increased method automation and an improved understanding of the interpretation of CMg outputs, including robust reporting thresholds to confirm the presence of pathogen species and AMR gene identifications.

**Supplementary Information:**

The online version contains supplementary material available at 10.1186/s12920-024-02044-w.

## Background

Wound infections, defined as the presence of proliferating microorganisms in viable tissue leading to a host immune response, tissue damage, and impaired wound healing [1], have a negative impact on the patient’s recovery and are associated with increased morbidity, mortality and healthcare costs [2–5]. Current diagnostics tests are limited by the turnaround time, suitability of the equipment for use in austere environments and the poor detection of fastidious and anaerobic organisms. Novel diagnostic approaches are required to reduce the turnaround time to enable wound infection diagnosis and inform antibiotic treatments, and in this study we considered the requirements of operating in austere environments outside of a fixed laboratory, including deployed military medical treatment facilities.

The skin is an integral barrier preventing microbes from contacting underlying tissues. When the skin barrier is broken or damaged, microbes originating from the body’s own flora, environmental contamination and nosocomial transmission [6, 7] contaminate and colonise the exposed tissue [1]. Depending upon the clinical context, microbial burden and the host immune response, the microbes can reach critical colonisation and cause a wound infection [8]. Identification of the causative pathogen and the associated AMR profile is key to reducing wound infection morbidity and mortality through improved antibiotic administration [9]. The use of broad-spectrum empirical antibiotics in the absence of conclusive identification of the organism is associated with the development of antibiotic resistance [10–12] making subsequent treatment more difficult [13]. In a military context, the significant and penetrating injuries resulting from battle mean that military personnel are especially susceptible to invasive bacterial and fungal wound infections [14, 15].

A range of factors, including the cause of the wound and the geographical location of the patient, influence the causative pathogen of an infection. *Pseudomonas aeruginosa* and *Staphylococcus aureus* are commonly identified in community and hospital-acquired wound infections in routine medical care, with elderly patients receiving wound care at home or in long-term care facilities being particularly susceptible to infection by *Streptococcus pyogenes* [12, 16–18]. An evaluation of combat related extremity wound infections from US personnel stationed in Iraq and Afghanistan identified *Acinetobacter* spp., *Escherichia coli*, *Pseudomonas* spp. and *Enterococcus* spp. as the most common cause of wound infections in this patient cohort [19]. The composition of wound bacterial isolates also changes over time with the incidence of poly-microbial and deep wound infections with AMR increasing in the second week post-injury [15, 20–22].

The identification of a suspected wound infection, including from acute and chronic wounds, is based upon clinical signs and symptoms, with diagnostic tests unlikely to be ordered until a spreading infection is suspected [1, 23]. Routine microbiological investigations and diagnostics involve culture-based isolation of microorganisms collected from the wound site, most commonly via a wound swab [24]. Microbial species are identified from isolated colonies using a range of tests including culture characteristics, specific selective and chromogenic agars, and analytical profile indexes (API). Additional methods including Matrix-assisted laser desorption/ionisation - time of flight mass spectrometry (MALDI-TOF-MS) and Polymerase Chain Reaction (PCR) are increasingly being utilised [25, 26]. Further antimicrobial susceptibility testing (AST) is carried out to inform the optimal antibiotic treatment [27]. Whilst microbiological culture is considered the gold standard, the turnaround time, requirement for large, static incubator equipment and poor sensitivity of detection of fastidious and anaerobic organisms [28] limits its use in military medical treatment facilities due to logistic and operative burdens.

CMg has the potential to provide a suitable alternative to culture-based diagnostics for a range of sample and infection types, including wound samples [29, 30]. The application of CMg for wound infections has so far been limited to individual case studies [31, 32]. Wound samples provide a challenge for CMg due to the variable causative pathogen species and the high microbial commensal flora and human cellular load associated with different wound types and mechanisms of injury [33]. Improvements in sample preparation, including the development of effective host DNA depletion methods to reduce the proportion of host genetic material and improve pathogen detection, alongside nanopore based DNA sequencing technology suitable for use in austere environments, mean that CMg can potentially identify any pathogen species and provide additional clinically relevant information within a rapid timeframe.

This study presents the development of a CMg workflow for wound swab samples followed by a prospective study analysing swab samples collected from patients with a suspected wound infection. An additional wound swab sample was collected from patients at two National Health Service (NHS) hospital trusts immediately following the collection of a sample for routine testing. The study sample was processed using the CMg workflow at the Defence Science and Technology Laboratory, Porton Down, and the results compared to routine microbiology testing. The workflow included host-DNA depletion, DNA extraction, whole genome amplification (WGA), library preparation and nanopore-based DNA sequencing steps followed by bioinformatic analysis of the sequence data. The overarching aim of the study was to demonstrate whether CMg provides a viable and enhanced diagnostic approach for wound infections and to inform on the parts of the workflow suitable for further optimisation to develop workflows for use in a low burden military diagnostic laboratory. CMg outputs from the prospective study were compared with hospital diagnostic laboratory culture results in addition to qPCR analysis for high priority wound pathogens.

## Methods

### Contrived sample generation

Prior to initiating the prospective clinical sample collections, the workflow was evaluated in contrived spiked samples using a saponin-based host depletion method previously developed for respiratory and whole blood samples [34, 35] and adapted here for wound swab samples. Contrived wound swab samples were generated by spiking blank Transwab^®^ with Amies Charcoal Agar (Medical Wire & Equipment) swabs with 50 µL whole human blood in dipotassium Ethylene Diamine Tetraacetic Acid collected from healthy donors (Cambridge Bioscience). Whole human blood was mixed with serial dilutions of clinically relevant bacteria strains ranging from 5.0 × 10^4^ to 5.0 × 10^2^ colony forming units (CFU) per swab. Spiked strains included *bla*CTX-M positive *E. coli* (National Collection of Type Cultures [NCTC] 13441) and *mecA* positive *S. aureus* (NCTC 14245). Cultures were grown in nutrient broth at 37 °C at 180 RPM for 16 h and quantified by plating in triplicate onto nutrient agar. Contrived samples were processed as follows.

### Pre-sequencing sample preparation

#### Host depletion

The swab tip was added to 200 µL phosphate-buffered saline (PBS) in a 2.0 mL tube. Forty microliters of 30% saponin (in PBS) was added with 200 µL heat labile salt activated nuclease (HLSAN) buffer (5.5 M NaCl and 100 mM MgCl_2_ in molecular-grade water) and 10 µL HLSAN DNase (ArcticZymes). The sample was incubated at 37 °C for 10 min at 1000 RPM on a thermomixer (Eppendorf). The sample was vortexed for 20 s at full speed and the sample was transferred to a new 2 mL tube and the swab tip disposed of. Nine hundred microliters of PBS was added and the sample was centrifuged at 12,000 × *g* for 3 min. The supernatant was discarded and the pellet was resuspended in 600 µL PBS. Negative controls, consisting of a blank swab processed alongside the participant swab samples were included prior to the host-depletion step.

#### DNA extraction

The resuspended pellet was transferred to a Matrix lysing E tube (MP Biomedicals) and bead beaten for 60 s at 6.0 m/s on a FastPrep-24™ 5G (MP Biomedicals) bead beater and centrifuged at 17,000 × *g* for 60 s. Four hundred microliters of clear supernatant was transferred to a new tube with 300 µL lysis buffer and 30 µL Proteinase K (Promega). The sample was incubated at 65 °C for 5 min at 1000 RPM and added directly to a MaxWell 16 LEV Blood cartridge (Promega). The DNA extraction was carried out by the MaxWell 16 LEV platform using the Blood DNA protocol and eluted into 50 µL elution buffer (Promega).

#### DNA quantification

Total DNA concentration was quantified using the double-stranded DNA high-sensitivity assay kit on the Qubit 4™ fluorometer (Invitrogen) according to the manufacturer’s instructions.

#### DNA purification

The DNA extract was purified by AMPure XP bead (Beckman Coulter) purification according to the manufacturer’s instructions at a ratio of 1.8 × beads to DNA extract in a 1.5 mL LoBind tube (Eppendorf) and eluted into 10 µL nuclease-free water.

#### Whole genome amplification

The purified DNA underwent WGA using the Illustra™ GenomiPhi™ V3 Ready-to-Go™ WGA kit (Cytiva). Ten microliters of purified DNA was added to 10 µL of 2 × denaturation buffer in a 0.2 mL PCR tube and heated to 95 °C for 3 min and cooled to 4 °C on ice. The 20 µL reaction was added directly to the freeze-dried reagent cake and incubated at 37 °C for 2 h followed by a heating step at 65 °C for 10 min. The sample was cooled to 4 °C on ice and the amplified DNA quantified as previously described.

#### DNA de-branching

Two microliters of NEBuffer and 1 µL of T7 Endonuclease (New England Biolabs) were added to a maximum of 1 µg amplified DNA in 17 µL total volume, adjusted with nuclease-free water. The sample was then incubated at 37 °C for 15 min. The de-branched DNA was quantified and purified using a 0.6 × ratio of AMPure XP beads to DNA extract, according to the manufacturer’s instructions.

### Nanopore DNA sequencing

#### Library preparation and MinION sequencing

The de-branched DNA was prepared for sequencing using the Rapid Barcoding (SQK-RBK004) library preparation kit (Oxford Nanopore Technologies) according to the manufacturer’s instructions. Five to seven samples plus a negative control were run per multiplexed sequencing run. Up to 400 ng of the DNA in a total volume of 7.5 µL was added to a 0.2 mL PCR tube. Two point five microliters of Fragmentation Mix RB01-12 was added, and the sample incubated at 30 °C for 1 min and 80 °C for 1 min and the sample was cooled briefly on ice. The DNA libraries were pooled into a 1.5 mL LoBind tube and mixed with an equal volume of AMPure XP beads. The pooled libraries underwent AMPure XP bead purification according to the manufacturer’s instructions and eluted into 10 µL of 10 mM Tris-HCl pH 7.5-8.0 with 50 mM NaCl. One microliter of the Rapid Adapter was added to 10 µL of the purified pooled libraries, and the sample was incubated at room temperature for 5 min. A R9.4.1 Flowcell (Oxford Nanopore Technologies) was inserted into an MK1c Sequencing device (Oxford Nanopore Technologies), and the flow cell was checked and primed for sequencing according to the manufacturer’s instructions. The pooled libraries were loaded according to the manufacturer’s instructions and sequenced for a total of 16 h, and the data was stored in Fast5 format.

#### Bioinformatic and statistical analysis

A bioinformatics pipeline for the taxonomic profiling of microbial species and for the identification of AMR genes, previously developed for the analysis of metagenomics datasets for infectious disease diagnostics, was applied [34]. Microbial species identification was presented as the total number of reads and the percentage of the total classified microbial reads aligned to the species. Species were concluded to be present if ≥ 5 reads, minus the number of reads of the same species identified in the negative control sample, matched that species. Alternative thresholds for considering a positive identification were applied to compare the sensitivity when these were applied, including ≥ 50 reads assigned to a species and the reads assigned to a species made up ≥ 0.1% of the classified microbial reads. Sequencing depth was calculated by dividing the total base pairs (bp) assigned to a species by the total genome size of the species. AMR genes were concluded to be present if their template identity and coverage were ≥ 90% and their depth of coverage reported by KMA were ≥ 1×. AMR genes were linked to the resistances reported by AST through the Comprehensive Antibiotic Resistance Database (CARD), which lists the species associated with specific AMR genes [36]. Bacterial species were grouped into aerobes, facultative anaerobes, and obligate anaerobes according to the Bac*Dive* database [37]. Data are expressed as means and standard deviation and were analysed using GraphPad Prism 10 (version 10.0.2) software. When multiple comparison analyses were performed, *p*-values were obtained by performing an ordinary one-way ANOVA test with a post hoc Tukey’s test. When two samples were individually compared, results were obtained using a 2-tail unpaired t-test. Sensitivity and specificity values were calculated according to the following calculations as previously described [38]:

$$Sensitivity\;=\left[\left(True\;Positives\right)/\left(True\;Positives\;+False\;Negatives\right)\right]\times100$$

$$Specificity\;=\left[\left(True\;Negatives\right)/\left(False\;Positives\;+\;True\;Negatives\right)\right]\times100$$

True positives = species identified by CMg and routine microbiology and/or qPCR.

False Negative = species identified by routine microbiology and/or qPCR but not identified by CMg.

True Negative = species not identified by CMg or routine microbiology and/or qPCR.

False positive = species identified by CMg but not by routine microbiology and/or qPCR.

Species α-diversity was calculated using the Shannon diversity index. The Shannon diversity index is an estimator for species richness and evenness [39]. The formula is as follows:

$$Shannon\;diversity\;index=-\sum\left[\left(pi\right)\times\ln\left(pi\right)\right]$$

$$pi=\frac nN$$

*n =* number of reads assigned to a microbial species in a sample.

*N =* total reads assigned to microbial species in a sample.

#### Ethical approval and sample collection

The study protocol for the prospective collection of wound swab samples, the provision of routine microbiology results, and previous antibiotic treatments from NHS patients with a suspected wound infection was approved by the Ministry of Defence Research Ethics Committee (MODREC) (Reference: 2149/MODREC/22). Participants with suspected wound infections from open wounds, defined as cuts, lacerations, abrasions, avulsions, punctures, bites, grades 2–4 pressure ulcers, arterial, venous or diabetic ulcers, and burns, were prospectively recruited from Salisbury District Hospital, UK (SDH) and Basingstoke and North Hampshire Hospital, UK (BNH). Closed wounds in which the skin barrier was not broken, such as those resulting from blunt trauma, were not included in the study. Written informed consent was obtained prior to sample collection. One additional wound swab sample was collected according to best practice [40] immediately following the collection of a standard of care wound swab. The additional swab sample was given an anonymised unique sample identifier and stored at 4–8 °C. Samples were processed for CMg analysis following the protocol described above.

#### Routine clinical microbiological investigation

Routine microbiology testing of the wound swab sample was carried out by NHS staff at each participating NHS trust according to standard protocols. Wound swabs were collected using a Transwab medium with charcoal (Amies) swab. Within 48 h of collection, wound swabs were streaked on to semi-solid agar media including Columbia Blood and Chocolate agar, incubated at 35–37 °C in 5% CO_2_; Columbia selective agar and Cysteine-, Lactose-, electrolyte deficient agar, incubated at 35–37 °C under aerobic conditions and Fastidious Anaerobe Agar with neomycin incubated at 35–37 °C under anaerobic conditions. Additional media, such as Sabouraud agar for the growth of mold and yeast species, were used for certain specific anatomical sites, or where particular clinical information or situations or specific clinician instruction determined their use based on the UK Standards for Microbiology Investigations (UK SMI) [40]. Plates were examined at 18–24 h and again at 48 h post-inoculation for growth with extended incubation if directed by the consultant microbiologist. Potentially significant isolates were selected and identified according to colony morphology, growth characteristics on particular media (e.g. haemolysis, growth on selective agar), Gram stain, and rapid biochemical tests. Confirmation of colony identity was carried out by MALDI-TOF-MS or by extended biochemical tests including API (BioMerieux). The level of identity reported was in accordance with the UK Standards for Microbiology Investigations [40] based on the anatomical site from where the swab was taken, the clinical presentation, the isolate, and following any specific clinical instructions.

AST was carried out by disc diffusion assay. Pure isolates were streaked on to Mueller-Hinton agar against antibiotic panels according to the isolate. Conduct of antibiotic susceptibility testing and interpretation of zones of inhibition was done according to European Committee On Antimicrobial Susceptibility Testing standards and guidelines [27].

#### Interpretation of CMg results

The interpretation of the sequencing results to identify the species considered to be reportable in a clinical context was carried out by a consultant microbiologist in the context of the UK Standards for Microbiology Investigations [40]. Species, including those that would only be reported in specific circumstances including when identified in isolation, were reported.

#### qPCR

TaqMan probe based qPCR assays were performed in duplicate for four high priority pathogen species. All assays were performed on the QuantStudio™ 7 Flex (ThermoFisher) platform, and quantitation cycle (Cq) values were determined by the QuantStudio™ software (ThermoFisher). Cq values below 38.0 were considered positive. Positive control reactions of purified bacterial DNA at a concentration of 1.0 ng/µL and negative control reactions of nuclease-free water only were run on each plate. The primer and TaqMan probe sequences of each assay are described in Table 1. The TaqMan Fast Advanced Master Mix (Applied Biosystems) was used for all assays. The following conditions were applied for each qPCR assay:

- *E. coli*: 25 µL reactions using 12.5 µL master mix, final concentrations of 900 nM forward primer, 900 nM reverse primer, 200 nM of probe, and 1 µL of template. Thermal cycling conditions were 1 min at 95 °C followed by 40 cycles of 15 s at 95 °C and 30 s at 60 °C [41].
- *P. aeruginosa*: 10 µL reactions using 5 µL master mix, 300 nM forward primer, 300 nM reverse primer, 200 nM TaqMan probe, and 1 µL template. Thermal cycling conditions were 1 min at 95 °C followed by 40 cycles of 15 s at 95 °C and 30 s at 60 °C [42].
- *S. aureus*: 10 µL reactions using 5 µL master mix, 1 µM forward primer, 1 µM reverse primer, 200 nM probe, and 1 µL template. Thermal cycling conditions were 1 min at 95 °C followed by 40 cycles of 15 s at 95 °C and 30 s at 57 °C [43].
- *S. pyogenes*: 25 µL reactions using 12.5 µL master mix, final concentrations of 100 nM forward and reverse primer, 150 nM probe and 1 µL template. Thermal cycling conditions were 1 min at 95 °C followed by 40 cycles of 20 s at 95 °C and 20 s at 60 °C [44].

**Table 1:** Primer and probe sequences for high priority pathogen qPCR assays

Assay target	Forward primer (5’-3’)	Reverse primer (5’-3’)	Probe sequence (5’-3’)	Source
*E. coli 16 S rRNA* gene	CATGCCGCGTGTATGAAGAA	CGGGTAACGTCAATGAGCAAA	TTCAGCGGGGAGGAAGGGAGTAAAGTTAATA	[41]
*P. aeruginosa oprL* gene	CAGGTCGGAGCTGTCGTACTC	ACCCGAACGCAGGCTATG	AGAAGGTGGTGATCGCACGCAGA	[42]
*S. aureus* protein coding sequence (NCBI Protein accession number: YP_500811.1)	AACTACTAGGGGAGCCTAATRAT	GGTACTAACCAAATCAGGTCATAA	TGGCTGAGATGAAYTGTTCAGACCC	[43]
*S. pyogenes speB* gene	CTAAACCCTTCAGCTCTTGGTACTG	TTGATGCCTACAACAGCACTTTG	CGGCGCAGGCGGCTTCAAC	[44]

## Results

### CMg wound sample method development

**Fig. 1:**
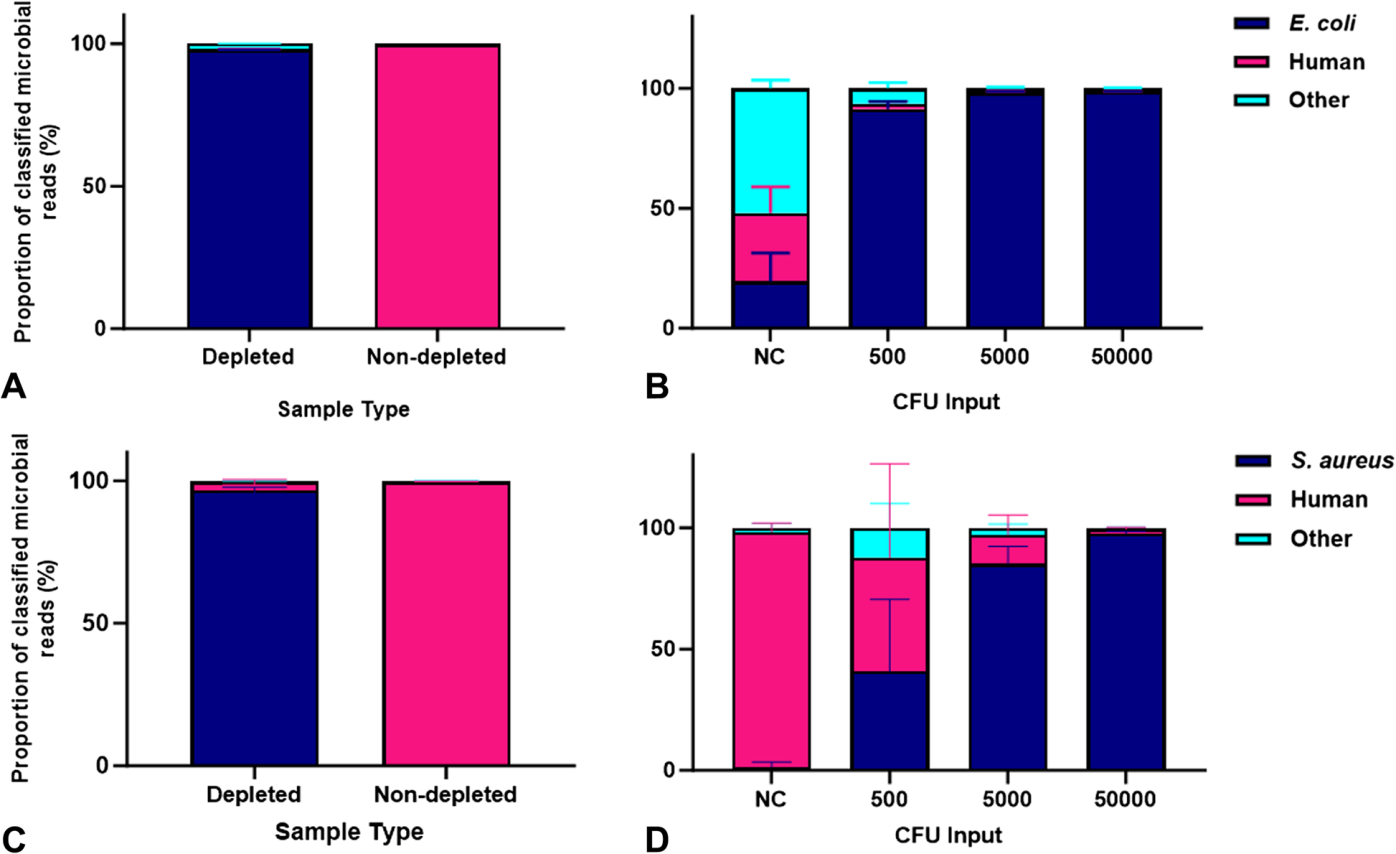
**A**-**D **Proportion of sequence reads assigned to spiked bacteria and human genomes from contrived wound swab samples. Blank wound swabs were spiked with *E. coli* (**A**) and *S. aureus* (**C**) and whole human blood and underwent the full CMg workflow. The proportion of total sequence reads assigned to the spiked bacteria and the human genome were compared to non-depleted control samples. Contrived samples were spiked with serial dilutions of *E. coli* (**B**) and *S. aureus* (**D**) (500–5.0 × 10^4^ CFU) and whole human blood and processed for CMg and the proportions of the total sequence reads were compared. +SD. NC = Negative control. *n* = 3

Initial testing of the workflow using contrived samples was carried out to investigate the appropriateness of the wound swab samples added to a charcoal growth media for CMg, including the effectiveness of the saponin-based host depletion step. When *E. coli* was included alongside human blood on a charcoal swab, the saponin-based host depletion protocol reduced the mean proportion of human DNA from 99.63 to 0.59% of total classified reads and increased the proportion of *E. coli* reads from 0.09 to 97.67% in the resulting sample metagenomes (Fig. 1A). In contrived wound swabs with *S. aureus*, host depletion reduced the mean proportion of human DNA from 99.73 to 2.87% and increased the mean proportion of *S. aureus* reads from 0.03 to 96.57% (Fig. 1C). In samples containing serial dilutions of *E. coli* from 5.0 × 10^4^ CFU to 500 CFU per swab, the mean proportion of *E. coli* reads remained high, decreasing from 98.41 to 91.27% (Fig. 1B). The mean proportion of *S. aureus* reads reduced from 97.56% in the 5.0 × 10^4^ CFU input to 40.77% in the 500 CFU input samples (Fig. 1D). The majority of non-spiked microbial reads present in the negative control samples were assigned to the fungal species *Aspergillus luchuensis*.

**Fig. 2:**
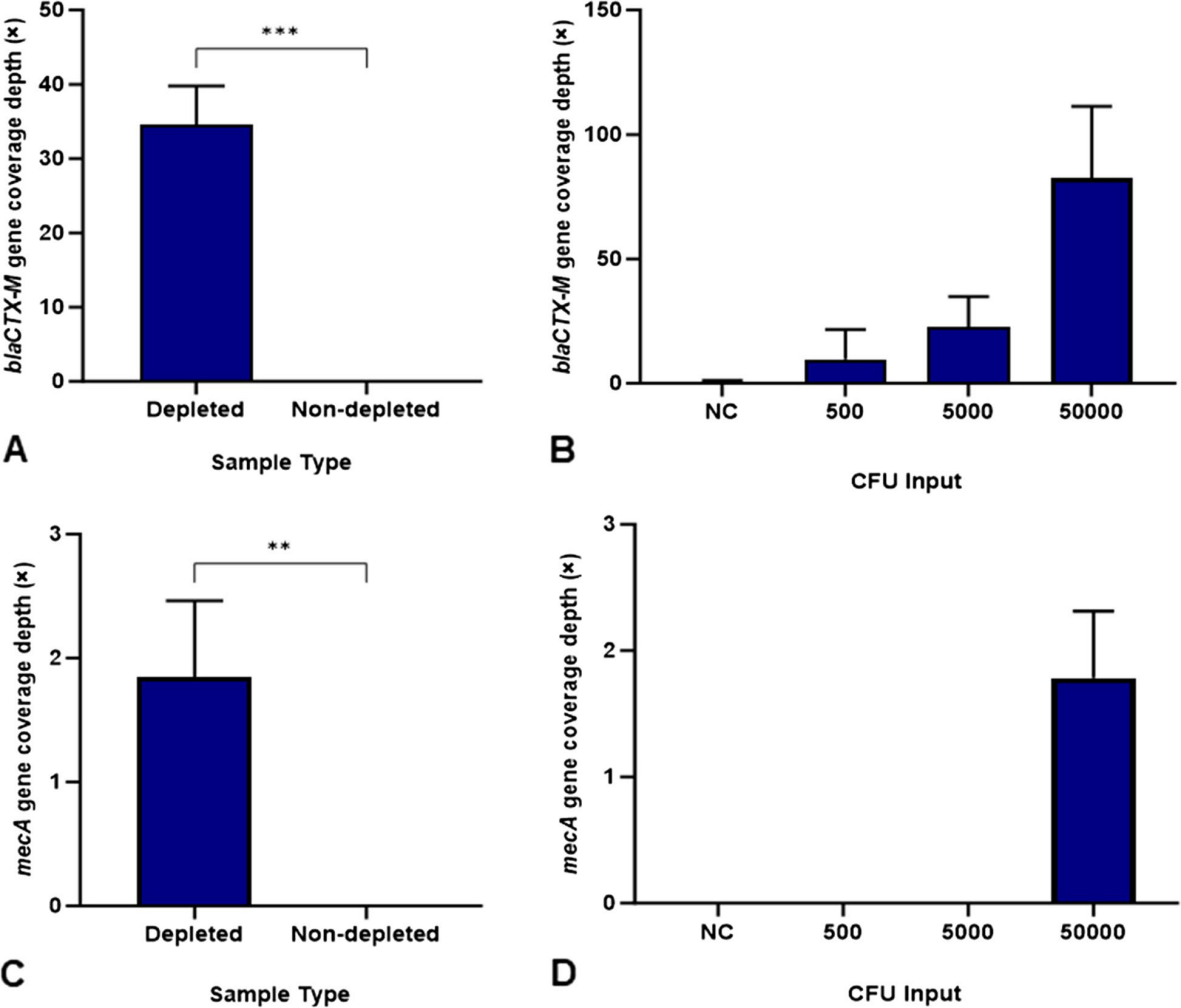
**A**-**D** Identification of AMR genes from contrived wound swab samples. The depth of AMR gene coverage (×) was measured from *E. coli* (**A**) and *S. aureus* (**C**) spiked samples compared to non-depleted control samples and from contrived samples spiked with serial dilutions of *E. coli* (**B**) and *S. aureus* (**D**). + SD. NC = Negative control. *n* = 3. ** *p* = < 0.01. ***p = < 0.001

The identification of AMR genes in the sample metagenomes was also compared. Host depletion of samples spiked with *E. coli* resulted in a significant increase in *bla*CTX-M gene coverage depth to 34.61× in depleted samples from 0× in the non-depleted samples (Fig. 2A). There was also a significant increase in coverage of the *mecA* gene from *S. aureus* spiked samples to 1.84× in the depleted sample from 0× in the non-depleted samples (Fig. 2C). From the spiked *E. coli* serial dilutions, *bla*CTX-M was identified at all spiked concentrations, ranging from 9.76× coverage in the 500 CFU input to 82.5 × in the 5.0 × 10^4^ CFU input (Fig. 2B). In samples spiked with serial dilutions of *S. aureus*, *mecA* was identified at the highest CFU input (5.0 × 10^4^ CFU) at 1.78× depth of coverage but was not identified at any other input (Fig. 2D).

### Prospective wound swab sample collection

Forty wound swab samples, prospectively collected from participants with suspected open wound infections, were processed for CMg and the results were compared to routine microbiology results obtained from a swab collected immediately prior. Samples were collected from wound types comprising of: 29 (72.50%) ulcers, 3 (7.50%) animal bites, 3 (7.50%) unspecified non-healing wounds, 2 (5.00%) pressure wounds and 1 (2.50%) each of burn, amputation and laceration wounds. Twenty one participants (52.50%) had previously been prescribed antibiotics and 15 participants (37.5%) were not prescribed antibiotics prior to sample collection. Antibiotic history was unknown for four (10%) participants. Full sample characteristics are presented in Additional File 1 Table 1.

### Sample processing for CMg

The mean total DNA concentration following the host-depletion and DNA extraction was 3.53 ng/µL, ranging from below the Qubit dsDNA HS assay limit of detection (0.05ng/µL) to 21.60 ng/µL. The mean total number of sequencing reads per sample was 105,688, with a mean number of classified microbial reads of 32,760 and mean number of human reads of 24,657, with a mean read length of 1249 bp. The host depletion, DNA extraction, WGA and library preparation steps were completed within approximately 4 h 15 min per batch of 5–7 samples and sequencing was carried out for a total of 16 h. The bioinformatic analysis pipeline required up to one hour to generate a report identifying the microbial species and AMR genes, producing a result approximately 21 h following the start of sample processing. Full sample processing results are provided in Additional File 1 Table 2.

### Routine microbiology

Routine microbiology testing, carried out by the NHS microbiology laboratories, reported the identification of a microorganism according to species, genus or phenotypic characteristic in 32 (80.00%) samples. Seventy one microorganisms were reported in total, with multiple microorganisms reported in 21 positive samples (Table 2). The remaining eight (20.00%) samples reported no microbial growth or mixed colonising organisms only without a microorganism species, genus or phenotypic characteristics reported. The pathogen identification and AST reports were provided within 48 h, usually as part of the same report, unless the pathogen represented a significant isolate with infection control consequences, which were reported at 24 h as an interim report. This included Group A *Streptococcus* and MRSA. Reports of mixed colonising organisms were reported at 24 h and no further AST was carried out. If no colonies were grown at 48 h then it was reported as “no growth”.

### CMg species identification

CMg identified a corresponding organism matching 62 of the 71 microorganism species, genus or phenotypic characteristics reported by routine microbiology testing at ≥ 5 reads, an overall sensitivity of 87% (Table 2). Additional organisms that would be considered reportable, as defined by consultant microbiologists reviewing the CMg results, were identified in 8/8 samples that reported no growth or mixed colonising organisms only by routine microbiology (≥ 5 CMg reads). Further, the reportable organisms included those identified at a high proportion of microbial reads (≥ 1% classified microbial reads) in 4/8 of these samples. From the 32 microbiology positive samples, CMg identified additional reportable pathogens at a high abundance (≥ 1% classified microbial reads) in 13 samples. This included high priority pathogen species *E. coli* in sample 38, *P. aeruginosa* in samples 30, 36, 38 and 40 and *S. pyogenes* in samples 10, 36, 37 and 39. CMg also identified the fungal pathogen *C. albicans* in sample 18.

**Table 2:** Results of routine microbiology testing and CMg analysis of wound swab samples

Sample Number	Routine microbiology report	Corresponding species identified by CMg	Reads (% classified microbial reads)	Sequencing Depth (×)	Additional reportable wound pathogens identified by CMg (≥1% classified microbial reads)	Reads (% classified microbial reads)	Sequencing Depth (×)
01	*Enterobacter cloacae*^a^	*Enterobacter hormaechei*	111837 (70.84)	29.76	*Enterobacter sp. DSM 30060*	21481 (13.61)	7.16
					*Enterobacter roggenkampii*	7014 (4.44)	1.04
					*E. cloacae complex sp. ECL414*	6398 (4.05)	0.22
					*Klebsiella pneumoniae*	1707 (1.08)	0.33
02	No growth reported	N/A	N/A	N/A	None identified	N/A	N/A
03	*S. aureus*	*S. aureus*	1555 (64.85)	0.39	*Finegoldia magna*^C^	231 (9.63)	0.06
04	*Pasteurella multocida*	*P. multocida*	2708 (31.25)	0.62	None identified	N/A	N/A
05	Mixed colonising organisms^b^	N/A	N/A	N/A	None identified	N/A	N/A
	*Enterococci*	*Enterococcus faecalis*	232570 (93.93)	93.48			
	Coliform^b^	*Klebsiella aerogenes*	8879 (3.59)	2.46			
06	Mixed colonising organisms^b^	N/A	N/A	N/A	None identified	N/A	N/A
07	*Streptococcus oralis*	*S. oralis*	3482 (22.49)	1.81	*Streptococcus constellatus*^C^	1030 (6.66)	0.45
	*E. cloacae*	Not identified	N/A	N/A	*Streptococcus milleri*^C^	716 (4.63)	0.54
					*Streptococcus anginosus*^C^	166 (1.07)	0.02
08	*E. coli*	*E. coli*	20441 (44.77)	5.19	*E. hormaechei*	9940 (21.81)	2.69
	*Klebsiella pneumoniae*	*K. pneumoniae*	4423 (9.70)	1.13	*E. cloacae*	1810 (3.97)	0.77
	*Enterococci*	*E. faecalis*	1839 (4.04)	0.67	*Citrobacter portucalensis*	954 (2.07)	495.75
					*F. magna*^C^	953 (2.07)	0.44
09	No growth reported	N/A	N/A	N/A	None identified	N/A	N/A
10	*S. aureus*	*S. aureus*	1126 (41.17)	0.39	*S. pyogenes*	1542 (56.31)	0.89
11	*S. aureus*	Not identified	N/A	N/A	None identified	N/A	N/A
	*Coagulase negative Staphylococcus*	Not identified	N/A	N/A			
	*Group D Streptococcus*	Not identified	N/A	N/A			
12	Coliform^b^	*Citrobacter koseri*	29984 (95.49)	6.89	None identified	N/A	N/A
13	Anaerobes^b^	*Bacteroides fragilis*	15092 (65.22)	2.29	*Streptococcus dysgalactiae*	2830 (12.23)	0.08
	Group A *Streptococci*	*S. pyogenes*	138 (0.60)	0.05	*F. magna*^C^	561 (2.42)	0.11
	*Enterococci*	*E. faecalis*	110 (0.48)	0.03			
	Coliform^b^	*C. koseri*	50 (0.22)	0.01			
14	*S. aureus*	*S. aureus*	23082 (99.84)	7.11	None identified	N/A	N/A
15	*S. aureus*	*S. aureus*	15365 (99.81)	4.92	None identified	N/A	N/A
16	*S. aureus*	*S. aureus*	1933 (33.40)	0.39	*F. magna*^C^	1880 (32.49)	0.46
17	Coliform^b^	*Klebsiella oxytoca*	68 (1.93)	0.01	*Pseudomonas* sp. NIBR-H-19	3127 (88.71)	0.76
	*E. faecalis*	Not identified	N/A	N/A			
	*Proteus *overgrowth	Not identified	N/A	N/A			
	Diptheroids^b^	*Corynebacterium striatum*	86 (2.44)	0.04			
18	*E. coli*	*E. coli*	35004 (66.03)	8.00	*Candida albicans*	3273 (6.17)	2.25
	Anaerobes^b^	*Lacticaseibacillus rhamnosus*	8859 (16.71)	1.68			
	Coagulase negative* Staphylococcus*	*Staphylococcus epidermidis*	537 (1.01)	0.18			
	Diptheroids^b^	*C. striatum*	2587 (4.88)	1.27			
19	Scanty growth coagulase negative* Staphylococcus*	Not identified	N/A	N/A	None identified	N/A	N/A
	Scanty growth Diptheroids^b^	*C. striatum*	1396 (93.19)	0.65			
20	*S. aureus*	*S. aureus*	186 (0.62)	0.05	None identified	N/A	N/A
	*Enterococcus*	*E. faecium*	161 (0.53)	0.05			
	Non-lactose fermenting coliform^b^	*E. coli*	23 (0.08)	0.00			
21	Group B *Streptococcus*	*Streptococcus agalactiae*	323 (0.48)	0.14	*C. koseri*	59255 (88.09)	12.93
	*S. aureus*	*S. aureus*	94 (0.14)	0.02	*Citrobacter sp. TBCP-5362*	3843 (5.71)	1.03
22	Alpha-haemolytic *Streptococcus*	*Streptococcus gordonii*	5522 (9.26)	3.43	*Morganella morganii*	45270 (75.88)	0.34
	*Enterococci*	*E. faecalis*	359 (0.60)	0.14			
	Coliform^b^	*E. coli*	73 (0.12)	0.02			
	Oxidase negative non-lactose fermenter^b^	*Proteus mirabilis*	18 (0.03)	0.00	*F. magna*^C^	914 (1.53)	0.18
	Diptheroids^b^	*C. striatum*	94 (0.16)	0.05			
23	Scanty growth Group C *Streptococcus*	*S. dysgalactiae*	280 (0.74)	0.02	None identified	N/A	N/A
	Scanty growth *S. aureus*	*S. aureus*	212 (0.56)	0.06			
	*Proteus*	*P. mirabilis*	28 (0.07)	0.01			
24	Yeasts^b^	*C. albicans*	3288 (38.97)	1.90	*E. faecalis*	2495 (29.57)	0.64
	Coagulase negative *Staphylococcus*	*Staphylococcus haemolyticus*	217 (2.57)	0.06			
25	Coagulase negative *Staphylococcus*	*S. epidermidis*	85 (0.45)	0.02	None identified	N/A	N/A
26	Group A* Streptococcus*	*S. pyogenes*	6994 (82.97)	3.10	None identified	N/A	N/A
	*S. aureus*	*S. aureus*	544 (6.45)	0.16			
27	*S. aureus*	*S. aureus*	868 (7.93)	0.17	*F. magna*^C^	1156 (10.56)	0.30
	Group G *Streptococcus*	*S. dysgalactiae*	6 (0.05)	0.00			
	*Diptheroids*^b^	*C. striatum*	5646 (51.55)	2.53			
28	*Pseudomonas*	*P. aeruginosa*	1294 (3.06)	0.20	*F. magna*^C^	1356 (3.21)	0.64
	Scanty Growth coagulase negative *Staphylococcus*	*Staphylococcus equorum*	14 (0.03)	0.00			
	*Proteus*	*P. mirabilis*	11 (0.03)	0.00			
	Diptheroids^b^	*C. striatum*	30433 (71.95)	15.63			
29	*S. aureus*	*S. aureus*	62 (65.26)	0.00	None identified	N/A	N/A
30	*E. faecalis*	*E. faecalis*	561 (0.46)	0.08	*P. aeruginosa*	101154 (82.80)	16.36
	*Strenotrophomonas maltophilia*	*S. maltophilia*	221 (0.18)	0.06	*Pseudomonas putida*	18103 (14.82)	3.87
	*Diptheroids*^b^	Not identified	N/A	N/A			
31	Mixed colonising organisms^b^	N/A	N/A	N/A	None identified	N/A	N/A
32	Group G *Streptococcus*	*S. dysgalactiae*	4783 (28.95)	0.27	*F. magna*^C^	320 (1.94)	0.14
	*Enterococcus*	*E. faecalis*	217 (1.31)	0.08			
	Coagulase negative *Staphylococcus*	Not identified	N/A	N/A			
33	Mixed colonising organisms^b^	N/A	N/A	N/A	*B. fragilis*^C^	70466 (73.74)	18.52
					*M. morganii*	10540 (11.03)	3.92
					*Peptoniphilus sp. SAHP1*^C^	3211 (3.36)	2.55
					*Bacteroides uniformis*^C^	2221 (2.32)	0.73
					*P. mirabilis*	1938 (2.03)	0.69
34	*Pseudomonas* spp.	*P. aeruginosa*	1224 (9.37)	0.17	None identified	N/A	N/A
35	Group C *Streptococcus*	*S. dysgalactiae*	1239 (6.86)	0.05	*E. faecalis*	1198 (6.63)	0.30
	*S. aureus*	*S. aureus*	252 (1.39)	0.05			
36	Group C *Streptococcus*	*S. dysgalactiae*	17990 (76.75)	0.69	*S. pyogenes*	631 (2.69)	0.10
	*S. aureus*	*S. aureus*	1377 (5.87)	0.32	*S. agalactiae*	547 (2.33)	0.10
					*P. aeruginosa*	314 (1.34)	0.05
37	Group C *Streptococcus*	*S. dysgalactiae*	20847 (76.04)	0.79	*Prevotella corporis*^C^	2089 (7.62)	1.15
					*B. fragilis*^C^	1180 (4.30)	0.17
	*S. aureus*	*S. aureus*	23 (0.08)	0.01	*S. pyogenes*	722 (2.63)	0.12
					*S. agalactiae*	452 (1.65)	0.08
					*F. magna*^C^	278 (1.01)	0.07
38	Mixed colonising organisms^b^	N/A	N/A	N/A	*Klebsiella michiganensis*	12527 (38.66)	2.31
					*S. anginosus*^C^	3261 (10.06)	1.02
					*K. oxytoca*	1667 (5.14)	0.14
					*K. pneumoniae*	1390 (4.29)	0.24
					*Citrobacter freundii*	1269 (3.91)	0.27
					*E. faecalis*	1182 (3.65)	0.30
					*B. fragilis*^C^	757 (2.33)	0.13
					*Klebsiella grimontii*	731 (2.25)	1.63
					*E. coli*	576 (1.78)	0.14
					*P. aeruginosa*	463 (1.43)	0.07
					*E. hormaechei*	442 (1.36)	0.03
					*M. morganii*	329 (1.01)	0.08
39	Mixed colonising organisms^b^	N/A	N/A	N/A	*F. magna*^C^	2946 (37.15)	0.54
					*Staphylococcus pettenkoferi*^C^	129 (1.63)	0.04
					*S. pyogenes*	103 (1.30)	0.03
					*S. agalactiae*^C^	97 (1.22)	0.03
40	Mixed colonising organisms^b^	N/A	N/A	N/A	*F. magna*^C^	4110 (18.01)	0.97
					*P. aeruginosa*	518 (2.27)	0.07

Species identified by routine microbiology and the corresponding species identified by CMg at the highest classified microbial read proportion are reported. Additional reportable pathogens identified by CMg, as determined by a consultant microbiologist, identified ≥ 1% classified microbial reads are listed. % classified microbial reads = % of species reads over the total number of taxonomically classified microbial reads obtained. Sequencing depth was calculated by dividing the total bp assigned to a species by the total genome size

^a^Routine microbiology test reporting refers to a group of *Enterobacter* species as *Enterobacter cloacae* for routine reporting purposes This group includes *Enterobacter hormaechei*, listed as the corresponding organism in sample 01

^b^Microbial species or genus identification not provided by routine microbiology report

^c^Organism reported by routine microbiology if identified in pure culture only

**Fig. 3:**
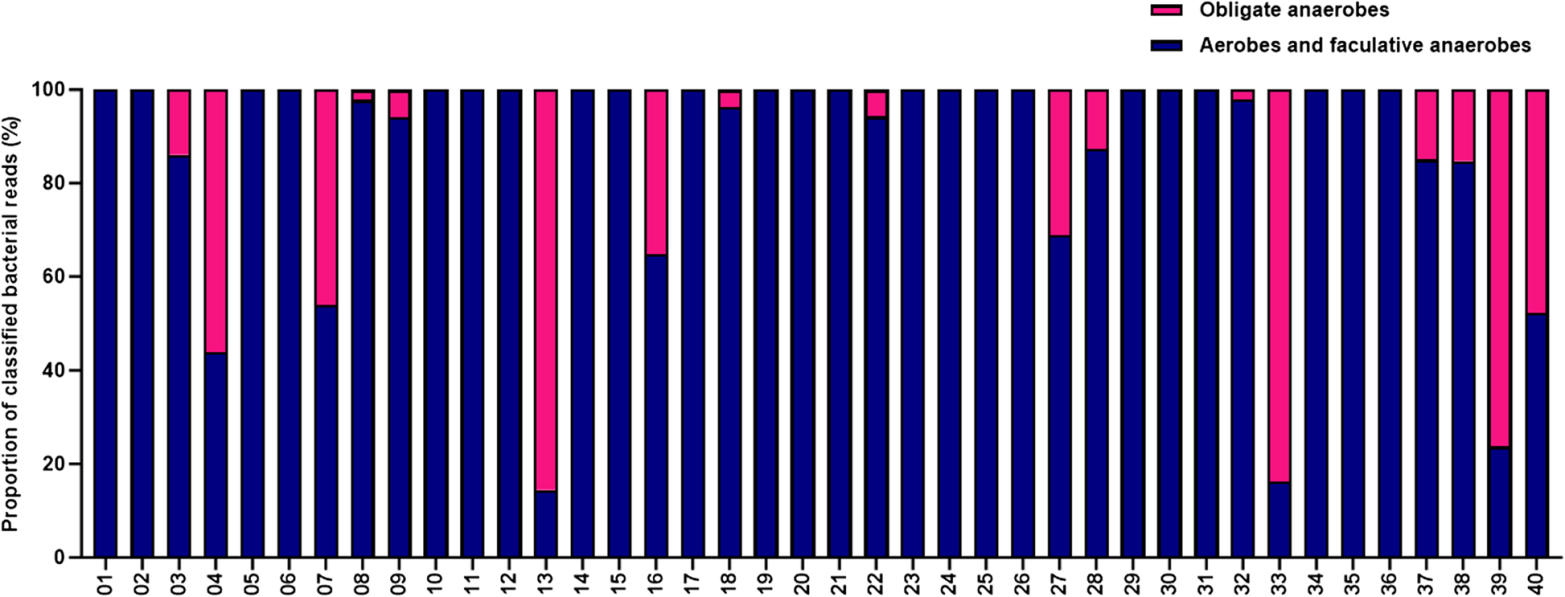
Proportion of aerobic and anaerobic bacteria species identified by CMg. All bacterial species identified at ≥ 1% of classified microbial reads were included and grouped as either obligate anaerobes or aerobes and facultative anaerobes and presented as the proportion of total bacterial reads

CMg reported the presence of obligate anaerobic species at a high proportion of microbial reads (≥ 1%) in 17 of the 40 samples (Fig. 3). The presence of anaerobic species was specifically reported by routine microbiology in two samples, 13 and 18. CMg reported the presence of anaerobic species in both of these samples. CMg reported high proportions of anaerobic species in several samples including 04, 07, 33, 39 and 40 in which routine microbiology did not specifically report the presence of anaerobes.

To provide additional corroboration of the CMg species identifications compared to microbiology testing, four high priority pathogens were selected for confirmatory testing by qPCR; *E. coli*, *S. aureus*, *P. aeruginosa* and *S. pyogenes*. The full list of qPCR Cq values and target species sequence reads and read lengths are provided in Additional File1 Table 3. To investigate the optimal threshold for the identification of a pathogen species by CMg from wound swab samples, the sensitivity and specificity of detection using three threshold values were compared. Applying the threshold of ≥ 5 reads assigned to a species for positive identification; CMg achieved a mean sensitivity of 83.82% and specificity of 66.64% across the four species (Table 3). Increasing the threshold to ≥ 50 reads reduced the sensitivity to 71.04% but increased the specificity to 90.66%. Applying a threshold of 0.1% of the total classified microbial reads achieved a sensitivity of 76.94% and a specificity of 82.85%. Modification of the thresholds had differing impacts on the sensitivity and specificity according to species. For example, the application of the 0.1% classified microbial read threshold or the ≥ 50 read threshold did not change the sensitivity of 80.00% for *S. aureus*. In contrast, this resulted in an increase in sensitivity for the detection of *P. aeruginosa* from 66.67 to 77.78%. Of the 24 samples that identified a high priority pathogen at a high concentration by qPCR (< 30 Cq), the pathogen was identified by CMg at ≥ 5 reads in 24 (100%) samples. This reduced to 21 (87.50%) and 22 (91.67%) when applying the ≥ 50 reads and 0.1% classified microbial read thresholds, respectively. The mean sequence read lengths of reads assigned to *S. aureus* and *S. pyogenes*, at 916 and 905 bp, respectively, were lower than both *E. coli* and *P. aeruginosa* at 1236 and 1474 bp, respectively.

**Table 3:** Sensitivity and specificity of CMg compared to qPCR and routine microbiology testing

Target species	Sensitivity (%)			Specificity (%)		
	≥ 5 reads	≥ 50 reads	0.1% classified microbial reads	≥ 5 reads	≥ 50 reads	0.1% classified microbial reads
*E. coli*	62.50	37.50	50.00	78.13	87.50	84.38
*P. aeruginosa*	77.78	66.67	77.78	87.10	96.77	96.77
*S. aureus*	95.00	80.00	80.00	50.00	100.00	80.00
*S. pyogenes*	100.00	100.00	100.00	51.35	78.38	70.27

Sensitivity and specificity of CMg compared to qPCR and microbiology testing for four high priority pathogen species according to three reporting thresholds; ≥5 reads assigned to the target species, ≥ 50 reads assigned to the species and 0.1% of the total classified microbial reads assigned to the target species. % classified microbial reads = % of species reads over the total number of taxonomically classified microbial reads obtained

**Fig. 4:**
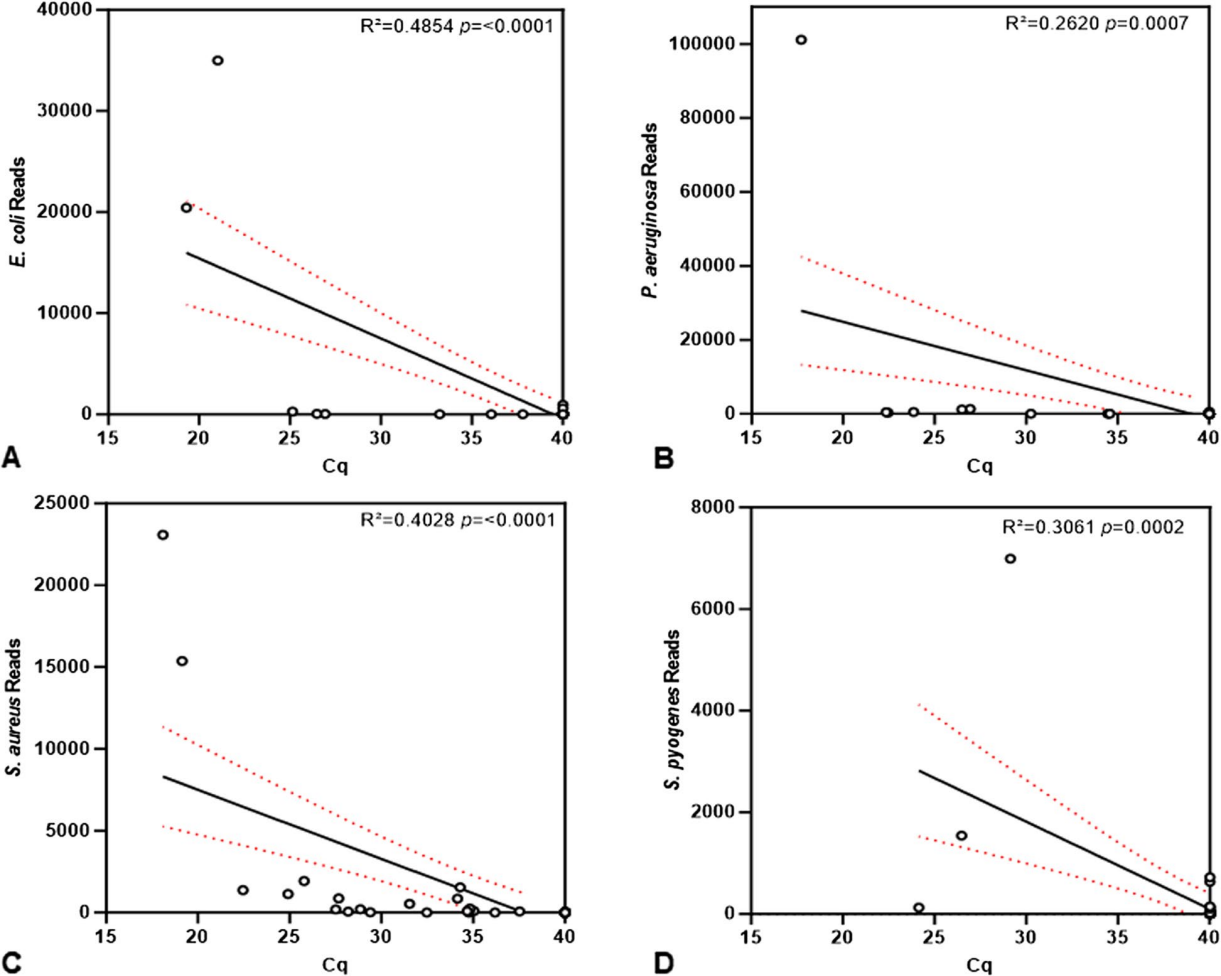
Correlation of qPCR with CMg for the detection of four high priority pathogen species. The qPCR Cq values and the number of reads assigned to *E. coli* (**A**) *P. aeruginosa* (**B**) *S. aureus* (**C**) and *S. pyogenes* (**D**) were compared, showing correlation for all four species. The linear regression line and 95% confidence interval are presented

To determine whether the number of sequencing reads assigned to a high priority pathogen represented the concentration of pathogen DNA in the DNA extract, the correlation of qPCR Cq values to the sequencing reads was calculated. There was a negative correlation between the Cq values and sequence reads for all four species, indicating that the number of reads assigned to the species was representative of the concentration of pathogen DNA in the sample (Fig. 4).

### Detection of AMR genes

AST was carried out on 34 colonies isolated from 24 samples. Of these 34 colonies, 20 reported resistance to one or more antibiotics and 14 were sensitive to all tested antibiotics. CMg reported the identification of ≥ 1 AMR genes corresponding to the organism and resistance profile identified by AST for 10 (50%) of the 20 resistant colonies. For example, *tetK*, associated with resistance to tetracycline and previously identified in *S. aureus* [45] was identified in sample 23, in which AST had identified *S. aureus* resistant to doxycycline. Across all samples, CMg identified ≥ 1 AMR genes in 32/40 samples (Table 4). AMR genes associated with resistance to macrolide antibiotics were most commonly identified with 48 AMR gene identifications, followed by streptogramins and lincosamides, with 47 and 44 AMR gene identifications, respectively.

**Table 4:** Results of routine microbiology AST and AMR genes identified by CMg from wound swab samples

Sample Number	AST Report	Antimicrobial Resistance genes identified by CMg sequencing	Template Coverage	Depth of coverage (×)	Associated antibiotic drug class resistance
01	*E. cloacae:* **Amoxicillin resistant**	*fosA*	100	23.75	Phosphonic acid
		***blaACT-23***	100	32.11	**Penam**, cephamycin, cephalosporin, carbapenem
02	No growth reported: AST not carried out.	None identified	N/A	N/A	N/A
03	*S. aureus*: Sensitive (Levofloxacin intermediate)	None identified	N/A	N/A	N/A
04	*P. multocida*: Sensitive	None identified	N/A	N/A	N/A
	*S. aureus*: Sensitive (Levofloxacin intermediate)				
05	Mixed colonising organisms: AST not carried out.	*blaCMY-2*	100	1	Penam, cephamycin, cephalosporin, carbapenem
		*vgaA*	100.25	2.05	Streptogramin, pleuromutilin, lincosamide
		*emrD*	103.04	1.47	Phenicol
		*qacR*	105.47	1.58	Fluoroquinolone
		*dfrS1*	100	1	Diaminopyrimidines
		*blaZ*	104.26	1.52	Penam
		*aac(6′)Ie*	104.1	1.82	Aminoglycoside
		*blaZ*	102.36	1.84	Penam
		*lsaA*	100	59.89	Streptogramin, lincosamide, pleuromutilin
		*vgaB*	98.07	1.64	Streptogramin, pleuromutilin
06	Mixed colonising organisms: AST not carried out.	None identified	N/A	N/A	N/A
07	*S. oralis*: Sensitive	*mefA*	100.08	5.33	Streptogramin, macrolide
		*msrD*	101.5	2.6	Streptogramin, macrolide
		*tetM*	100.16	1.79	Tetracycline
	*E. cloacae*: Amoxicillin resistant	*tetL*	99.71	3.84	Tetracycline
		*ermF*	100	5.19	Streptogramin, macrolide, lincosamide
		*blaCFXA3*	100.21	2.81	Cephamycin
08	*E. coli: *Sensitive	*erm*X	100.23	3.68	Streptogramin, macrolide, lincosamide
		*tet*D	101.35	1.8	Tetracycline
		*kdeA*	99.59	2.36	Multidrug resistance
		*acrF*	100	8.96	Fluoroquinolone, penam, cephamycin, cephalosporin
	*K. pneumoniae*: **Amoxicillin resistant**	*erm*A	102.73	1.81	Streptogramin, macrolide, lincosamide
		*aph*(3')-IIIa	100.25	2.72	Aminoglycoside
		*erm*B	100.95	2.01	Streptogramin, macrolide, lincosamide
		*oqxA*	100.43	2.88	Tetracycline, diaminopyrimidine, glycylcycline, nitrofuran, fluoroquinolone
		*bla*CFXA	95.34	1.6	Oxacephem, cephamycin
	*Enterococcus* species: AST not carried out.	*emr*E	100	9.96	Multidrug resistance
		*bla*EC	100.09	5.63	Cephalosporin
		*fos*A2	101.41	2.01	Phosphonic acid
		***bla*ACT**	100.17	3.03	Carbapenem, cephalosporin, cephamycin, **penam**
		*emrD*	99.16	2.55	Phenicol
09	Mixed colonising organisms: AST not carried out.	None identified	N/A	N/A	N/A
10	*S. aureus*: Sensitive	*tet38*	100.23	3.68	Tetracycline
		*blaZ*	101.35	1.8	Penam
11	*S. aureus*: Sensitive	None identified	N/A	N/A	N/A
	Coagulase negative *Staphylococcus*: AST not carried out				
	Group D *Streptococcus*: AST not carried out				
12	Coliform: **Amoxicillin resistant**	***blaCKO***	100	8.46	**Penam**
13	Group A *Streptococci*: **Tetracycline resistant**	***tetM***	100	3.73	**Tetracycline**
		*cepA*	100	7.61	Cephalosporin
14	*S. aureus*: Levofloxacin resistant	*mepA*	100.07	4.69	Tetracycline, glycylcycline
		*blaZ*	100	25.67	Penam
		*blaR1*	100	31.91	Penam
		*tet38*	100	5.47	Tetracycline
		*lmrS*	100	55.75	Aminoglycoside, macrolide, phenicol, diaminopyrimidine, oxazolidinone
15	*S. aureus*: Levofloxacin resistant	*mepA*	100	9.77	Tetracycline, glycylcycline
		*blaZ*	100	29.72	Penam
		*blaR1*	100	28.4	Penam
		*tet38*	100	8.48	Tetracycline
		*lmrS*	103.19	3.82	Aminoglycoside, macrolide, phenicol, diaminopyrimidine, oxazolidinone
16	*S. aureus*: Levofloxacin resistant	*fos*B	100.95	2.01	Phosphonic acid
17	AST not carried out.	None identified	N/A	N/A	N/A
18	*E. coli:* **Amoxicillin, CoTrimoxazole, Ceftriaxone**, **CoAmoxiclav**, **Cefpodoxime**, **Gentamicin resistant**	***dfrA17***	100	24.01	**Diaminopyrimidine**
		***Sul1***	100	14.14	**Sulfonamide**
		*vgaA*	100.25	1.45	Streptogramin, pleuromutilin, lincosamide
		***acrF***	99.97	18.44	Fluoroquinolone, **penam**, cephamycin, **cephalosporin**
		*mdtM*	98.95	14.4	Nucleoside, fluoroquinolone, phenicol, lincosamide
		***blaTEM***	100	14.55	**Penam**, monobactam, **cephalosporin**, penem
		*mphA*	100	25.32	Macrolide
		***aph(6)-Id***	100	26.15	**Aminoglycoside**
		***aadA5***	100	16.61	**Aminoglycoside**
		***aac(3)-IId***	100	30.91	**Aminoglycoside**
		*tetW*	100.21	4.53	Tetracycline
		*tetA*	100	21.1	Tetracycline
		***sul2***	100	26.14	**Sulfonamide**
		***blaCTX-M***	100	5.88	**Cephalosporin**
		*emrE*	100	27.67	Multidrug resistance
		*ermX*	100	2.48	Streptogramin, macrolide, lincosamide
		***aph(3'')-Ib***	99.88	26.74	**Aminoglycoside**
		***blaEC***	100	16.08	**Cephalosporin**
		*emrD*	100	17.66	Phenicol
19	AST not carried out.	*sul1*	103.5	2.03	Sulfonamide
		*ermX*	100	3	Streptogramin, macrolide, lincosamide
20	*S. aureus*: **Erythromycin**, **Clindamycin resistant**	*ermX*	99.88	6.46	Streptogramin, macrolide, lincosamide
		*tetW*	100	114.6	Tetracycline
		*qacR*	101.59	2.01	Fluoroquinolone
		*dfrS1*	103.7	1.96	Diaminopyrimidines
		*sul1*	100	71.52	Sulfonamide
		*ant(2'')-Ia*	100	133.1	Aminoglycoside
		*aph(6)-Id*	101.91	2.02	Aminoglycoside
		*tetK*	100	16.92	Tetracycline
		***ermC***	100.14	3.62	Streptogramin, **macrolide**, lincosamide
		*tet33*	100	128.19	Tetracycline
		*ermX*	100	25.8	Streptogramin, macrolide, lincosamide
		*ermX*	100.13	256.3	Streptogramin, macrolide, lincosamide
21	Group B *Streptococcus*: Sensitive	*tetM*	99.69	1.19	Tetracycline
		*blaMAL-1*	100	34.79	Carbapenem
		*tetK*	100	22.67	Tetracycline
	*S. aureus*: Sensitive	*ermC*	100	35.48	Streptogramin, macrolide, lincosamide
		*spd*	100	52.6	Aminoglycoside
		*fosA7.3*	100	13.21	Phosphonic acid
22	AST not carried out.	*blaDHA-5*	100	27.76	Cephamycin, cephalosporin
		*mefA*	101.07	2.01	Streptogramin, macrolide
		*ermA*	100	4.45	Streptogramin, macrolide, lincosamide
		*catA2*	99.84	24.59	Phenicol
		*ant(6)-Ia*	101.87	1.94	Aminoglycoside
23	*S. aureus*: **Doxycycline**, **Erythromycin**, **Clindamycin**, Fusidic acid, Norfloxacin resistant	***tetK***	100	7.64	**Tetracycline**
		*tetW*	100	67.76	Tetracycline
		***ermA***	107.1	2.06	Streptogramin, **macrolide**, **lincosamide**
	Group G *Streptococcus*: Doxycycline resistant	*ermX*	100.13	16.3	Streptogramin, macrolide, lincosamide
		*aac(3)-XI*	100	6	Aminoglycoside
24	AST not carried out.	*dfrC*	100	9.28	Diaminopyrimidine
		*sul1*	100.71	2.01	Sulfonamide
		*tet(W)*	100	4.74	Tetracycline
		*ermC*	100	5.04	Streptogramin, macrolide, lincosamide
		*aac(6')-Ie*	100.07	6.17	Aminoglycoside
		*ermB*	100.41	2.79	Streptogramin, macrolide, lincosamide
25	AST not carried out.	*dfrC*	100	2	Diaminopyrimidine
		*sat4*	100	8.83	Nucleoside
		*blaZ*	100	11.2	Penam
		*ermB*	100	4.8	Streptogramin, macrolide, lincosamide
		*blaR1*	100.06	6.36	Penam
		*aph(3')-IIIa*	100	4.29	Aminoglycoside
		*ant(6)-Ia*	100	6.18	Aminoglycoside
		*blaZ*	100	5.8	Penam
26	*S. aureus*: Norfloxacin resistant	*blaTEM*	100	3	Penam, monobactam, cephalosporin, penem
	Group A *Streptococcus*: **Doxycycline resistant**	***tetM***	100	6.12	**Tetracycline**
27	*S. aureus*: Doxycycline, **Erythromycin**, **Clindamycin resistant**	*ermX*	100	11.97	Streptogramin, macrolide, lincosamide
		*blaTEM*	102.67	1.84	Penam, monobactam, cephalosporin, penem
		***ermA***	100	5.73	Streptogramin, **macrolide, lincosamide**
	Group G *Streptococcus*: Doxycycline resistant	*ermX*	100.13	109.45	Streptogramin, macrolide, lincosamide
		*tetW*	100	46.8	Tetracycline
28	AST not carried out.	*ermX*	100	7	Streptogramin, macrolide, lincosamide
		*iri*	100	2	Rifamycin
		*tetM*	100.16	4.14	Tetracycline
		*sat4*	100.18	6.01	Nucleoside
		*tetW*	100	33.01	Tetracycline
		*ermA*	100	25.91	Streptogramin, macrolide, lincosamide
		*aph(3')-IIIa*	100	5.81	Aminoglycoside
		*ant(6)-Ia*	100	2.89	Aminoglycoside
		*cmx*	102.47	2.34	Phenicol
		*ermX*	100.13	15.15	Streptogramin, macrolide, lincosamide
		*ermX*	98.83	1.71	Streptogramin, macrolide, lincosamide
		*crpP*	100.51	2.01	Fluoroquinolone
		*aac(3)-XI*	100	6.78	Aminoglycoside
29	*S. aureus*: Sensitive	None identified	N/A	N/A	N/A
30	AST not carried out.	*blaPDC*	100	2.28	Monobactam, carbapenem, cephalosporin
		*blaOXA-50*	100	27.1	Penam, cephalosporin
		*mexA*	100	46.28	Peptide, sulfonamide, diaminopyrimidine, phenicol, aminocoumarin, tetracycline, penam, cephalosporin, carbapenem, monobactam, fluoroquinolone, macrolide, penem, cephamycin
		*fosA*	100	12.8	Phosphonic acid
		*catB7*	100	19.85	Phenicol
		*aph(3')-IIb*	100.12	3.9	Aminoglycoside
		*mexX*	100	6.54	Aminoglycoside, phenicol, tetracycline, carbapenem, fluoroquinolone, macrolide, penam, cephamycin, cephalosporin
		*mexE*	100	7.04	Diaminopyrimidine, phenicol, fluoroquinolone
31	Mixed colonising organisms: AST not carried out.	*ermC*	105.03	1.94	Streptogramin, macrolide, lincosamide
32	Group G *Streptococcus*: **Doxycycline resistant**	***tetM***	99.69	2.64	Tetracycline
		*tetW*	100	12.42	Tetracycline
		*ermX*	100.13	8.34	Streptogramin, macrolide, lincosamide
33	Mixed colonising organisms: AST not carried out.	*tetD*	100	6.72	Tetracycline
		*blaDHA*	100	7.49	Cephalosporin, cephamycin
		*sat4*	100.37	2	Nucleoside
		*tetW*	100	12.67	Tetracycline
		*tetM*	100.16	3.63	Tetracycline
		*ermA*	100.82	2.01	Streptogramin, macrolide, lincosamide
		*aph(3')-IIIa*	100.5	2.01	Aminoglycoside
		*tetQ*	100	14.38	Tetracycline
		*tetJ*	100.33	1.96	Tetracycline
		*blaCFXA3*	100	405.12	Cephamycin
		*cepA*	100	27.96	Cephalosporin
		*ermX*	100.13	12.35	Streptogramin, macrolide, lincosamide
		*aac(3)-XI*	100	1	Aminoglycoside
34	*Pseudomonas *spp.: Sensitive (ciprofloxacin, ceftazidime, piperacillin-tazobactam, gentamicin intermediate)	*tetW*	100	15.68	Tetracycline
		*ermX*	100.13	6.92	Streptogramin, macrolide, lincosamide
35	Group C *Streptococcus*: erythromycin, clindamycin resistant	*qacB*	101.94	1.41	Fluoroquinolone
		*dfrS1*	100	5.51	Diaminopyrimidine
		*tetW*	100	5.95	Tetracycline
		*blaZ*	100	8.44	Penam
		*blaR1*	101.37	2.67	Penam
		*qacC*	100	11.83	Fluoroquinolone
	*S. aureus*: Sensitive (levofloxacin intermediate)	*blaZ*	100	8.12	Penam
		*bcrC*	100	3	Peptide
		*bcrB*	100	5	Peptide
		*ermX*	100.13	10.37	Streptogramin, macrolide, lincosamide
		*aac(3)-XI*	100	3	Aminoglycoside
36	Group C *Streptococcus*: erythromycin, clindamycin resistant	*ermX*	100.23	1.66	Streptogramin, macrolide, lincosamide
		*ermA*	101.23	1.01	Streptogramin, macrolide, lincosamide
		*fosB*	100.71	2	Phosphonic acid
	*S. aureus*: Sensitive (levofloxacin intermediate)	*catB7*	100.31	1.84	Aminoglycoside
		*mepA*	101.18	2.33	Tetracycline, glycylcycline
37	Group C *Streptococcus*: erythromycin, clindamycin resistant	*ermA*	100.27	1.84	Streptogramin, macrolide, lincosamide
	*S. aureus*: Sensitive (levofloxacin intermediate)				
38	Mixed colonising organisms: AST not carried out.	*tetM*	101.09	3.99	Tetracycline
		*blaOXY-1-7*	101.35	9.84	Penam, cephalosporin, monobactam
		*tetW*	99.96	2.02	Tetracycline
		*aph(3')-IIIa*	100.68	1.8	Aminoglycoside
		*ant(6)-Ia*	101.64	2.01	Aminoglycoside
		*aad9*	100.99	15.95	Aminoglycoside
		*ermF*	100	16.62	Streptogramin, macrolide, lincosamide
39	Mixed colonising organisms: AST not carried out.	*ermA*	100	5.74	Streptogramin, macrolide, lincosamide
		*ermC*	100	14.53	Streptogramin, macrolide, lincosamide
		*qacC*	100	41.27	Fluoroquinolone
		*ermX*	100.7	2	Streptogramin, macrolide, lincosamide
		*qacG*	100	1	Fluoroquinolone
		*tetM*	100.52	2.75	Tetracycline
40	Mixed colonising organisms: AST not carried out.	*ermX*	100	20.74	Streptogramin, macrolide, lincosamide
		*tetM*	100	5.49	Tetracycline
		*ermA*	100	14.48	Streptogramin, macrolide, lincosamide
		*ermC*	100	17.01	Streptogramin, macrolide, lincosamide
		*qacC*	100	17.6	Fluoroquinolone
		*tet33*	97.55	1.17	Tetracycline
		*qacG*	100	2.98	Fluoroquinolone

Results of routine microbiology AST from each routine wound swab and the AMR genes identified from the corresponding additional wound swab sample are reported. The antibiotic class to which each AMR gene is associated, according to the CARD is reported. AMR genes associated with resistance to antibiotic drug classes identified by AST in the same sample are highlighted in bold

### Species diversity

**Fig. 5:**
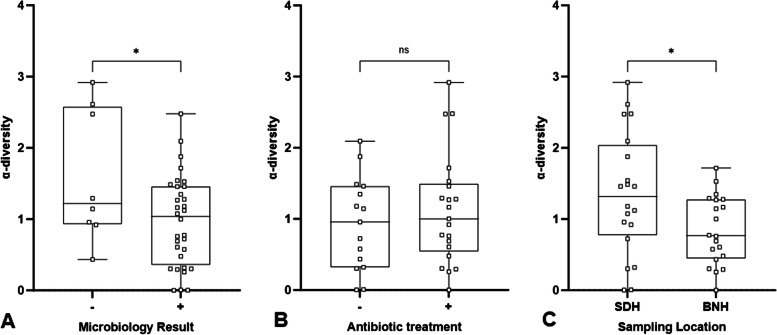
Comparison of microbial species α-diversity. The α-diversity, determined by calculating the Shannon diversity index for each sample, was compared between samples that tested positive and negative by routine microbiology testing (**A**), antibiotic treatment prior to sample collection (**B**) and wound swab sampling location (**C**). Error bars represent the minimum and maximum values. ns *p* > 0.05. *p = < 0.05. Microbiology and sampling location *n* = 40. Antibiotic treatment *n* = 36. The antibiotic history of four participants was unknown and these samples were omitted from the antibiotic treatment comparison. SDH = Salisbury district hospital. BNH = Basingstoke North Hospital

The species α-diversity in CMg data sets from each sample was determined by calculating the Shannon diversity index. There was a reduction in the α-diversity from samples in which routine microbiology testing reported the presence of a colony according to species, genus or phenotypic characteristic compared to samples that reported no growth or colonising microorganisms only (Fig. 5A). There was also a reduction in the species α-diversity associated with the sampling location, with the species diversity lower in samples collected at BNH compared to SDH (Fig. 5C). However, there was no change in α-diversity observed between patients that had undergone antibiotic treatment prior to sample collection (Fig. 5B).

## Discussion

Microbiology based diagnostic tests are well established for use in fixed laboratories and enable a plethora of tests to identify the causative pathogens and inform treatment. As such, these methods continue to be the gold standard for wound infection diagnosis. However, the equipment, time and biomedical scientist expertise required to grow and identify the microorganisms, particularly for fastidious and fungal species, mean that there are opportunities to develop additional improved methods. This is critical for austere environments such as deployed military medical treatment facilities in which time and physical space are at a premium. CMg offers a potential alternative to microbiology based testing and has been applied to numerous sample types, including respiratory, blood and cerebrospinal fluid [30, 46, 47]. This study presents the development of a CMg workflow that identifies reportable wound pathogen species with good concordance to routine microbiology testing and qPCR. Wound swab samples were processed directly without the need for a growth step, removing the requirement for incubator equipment and reducing the turnaround time, reaching the sequencing step in approximately four hours, completing sequencing within 20 h and the data analysis within 21 h of sample receipt, and enabling untargeted pathogen detection. The identification of AMR genes, key to informing rapid antimicrobial treatment, was demonstrated and additional data provided by CMg, including the α-diversity, could be applied to improve the clinical interpretation of CMg outputs. Additional studies applying CMg to wound swab samples and further method optimisation focused on increased automation are required to inform clinical interpretation and to enable the routine use of CMg for wound infection diagnosis in austere environments.

Contrived spiked swab samples were used to generate replicable samples with known bacterial and human cellular input. As previously demonstrated for other sample types, application of the saponin-based host depletion method resulted in the efficient depletion of human DNA and significantly increased the proportion of spiked bacterial sequence reads, showing the compatibility of the methods with the wound swab sample type prior to the collection of patient samples. However, the proportion of *S. aureus* reads and AMR gene coverage depth at the lowest bacteria input was lower compared to *E. coli*. This may be due to inherent bias introduced during the CMg workflow, including the DNA extraction and WGA steps [48, 49]. The reduced depth of coverage of the *mecA* gene may also be attributed to the chromosomal location of the staphylococcal cassette chromosome containing the *mecA* gene [50] compared to the *blaCTX-M* gene, located on a plasmid [51], potentially impacting the number of AMR gene copies added at each CFU input. However, despite the reduced proportion of *S. aureus* reads in contrived samples, the CMg sensitivity of *S. aureus* detection in patient samples was higher compared to *E. coli*, suggesting that the detection of *S. aureus* was not impaired by possible method biases. Notably, during method development, contrived samples were processed immediately following spiking whilst the participant samples were processed following storage at 4–8 °C. The storage of the wound samples in the presence of antibiotics, which empirically target gram-positive organisms alongside the host immune cells may have caused the *S. aureus* cells to be more susceptible to cell lysis, improving the DNA extraction efficiency and reducing the bias observed during method development. These results highlighted the importance of further understanding the species and AMR gene detection bias to improve the interpretation of CMg outputs. Further optimisation of the workflow should include evaluation of additional pathogen species, including high priority pathogen species and fungal species, to determine the impact of any introduced bias on the detection of these species.

Once the CMg workflow had been demonstrated in contrived samples, 40 wound swab samples were prospectively collected from patients at two NHS hospitals for CMg analysis. Consistent with previous studies [52–54], CMg identified a greater diversity of species than was reported by routine microbiology, including additional reportable pathogen species in microbiology negative samples, fungal species and anaerobic species. Routine microbiology is selective in nature, with the selection of the growth media, evaluation of plates by biomedical scientists and reports to clinicians all focused on the pathogen species relevant to the clinical context of the patient [40]. For example, anaerobic species were identified in several samples by CMg in which they were not reported by routine microbiology. Anaerobic species are only tested for according to the sample type, including wound swabs of traumatic wounds or swabs of pus. As such, the additional identification of anaerobic species by CMg may not reflect the detection of additional anaerobic species, but that routine microbiology did not test for these species at all. CMg therefore represents an opportunity to improve our understanding of the presence of anaerobic species in wound infections. However, it should be noted that the presence of anaerobic species revealed by CMg does not necessarily mean that these species are the cause of the infection and therefore require treatment. Further research is first required to understand the consequences of the presence of anaerobic species at wound sites and the associated clinical outcomes.

Further to this, pathogen species may be ignored if not considered relevant or missed entirely if cultured with morphologically similar colonising species. In keeping with the UK Standards for Microbiology recommendations, microbiology reporting may only specify the genus or phenotypic characteristics of a microorganism, further complicating the interpretation of CMg results. In addition, certain microbial species may be present on the wound but not culturable under the specific growth conditions employed during routine testing, which may explain the discordance between CMg and routine microbiology reports observed in this study. Therefore, to understand further the CMg outputs in relation to microbiology testing, four high priority pathogen species were selected for species-specific qPCR testing. There were examples of non-concordant results between culture, qPCR and CMg. Species misidentifications are a common issue for metagenomics studies, especially when pathogen species may share similar genomic regions to commensal species also present in high abundance. In this study there were samples, such as sample 01, in which CMg identified a high number of species reads that were not confirmed by culture or qPCR. To minimise these false-positive identifications, previous CMg studies have applied reporting thresholds, such as the total number of reads or the proportion of microbial reads assigned to a species. A reporting threshold of ≥ 5 reads assigned to a species, applied in this study, resulted in an average sensitivity of 83.82% and specificity of 66.64% across the four high priority species. Application of the reporting thresholds of ≥ 50 reads and 0.1% of classified microbial reads increased the specificity for all tested species. However, changing the reporting thresholds had different impacts on each tested high priority species, due to the higher proportion of false-positive samples for *S. aureus* and *S. pyogenes* with < 50 reads, resulting in increased specificity observed in these species when this threshold was applied, as an example. Importantly, when compared to qPCR alone, species identified with a Cq < 30 were reliably identified by CMg, with the sensitivity ranging from 87.50 to 100% depending on the CMg reporting threshold applied. An improved understanding of the interpretation of the identification of wound pathogens, including the relationship between the proportions/total number of sequence reads assigned to a specific pathogen and the cause of the wound infection, is required. This should include the development of a comprehensive list of wound pathogens and statistically robust reporting thresholds, developed by the scientific and clinical community, for optimal sensitivity and specificity. Development of these reporting thresholds would aid in the interpretation of CMg to inform infection treatment. The output of CMg workflows can include a myriad of microbial species of variable relevance to the treatment of the infection. Improved understanding of the consequences of the identification of each species and whether the treatment should be adjusted accordingly will be key to the implementation of CMg for wound infections.

Invasive fungal wound infections have been reported amongst military personnel [55]. The identification of fungal species by routine microbiology requires culturing on additional growth media, adding to the turnaround time and the resource requirements. The result may only then report the presence of yeasts without providing a species-specific identification, as with sample 24 in this study. The CMg workflow enabled the identification of fungal pathogens at the species level, including in sample 18 where routine microbiology did not report the presence of a fungal species at all, as the sample was not tested for fungal species. The rapid identification of fungal species without the need for additional testing could enable clinicians to target fungal pathogens faster, reducing the spread of invasive fungal wound infections.

The untargeted nature of CMg enables the provision of additional clinically relevant information. One metric that could be applied in a clinical context is the diversity of microbial species present at a wound site. There was a significant reduction in the species α-diversity between samples that tested positive by routine microbiology testing compared to negative samples. Previous studies have linked bacterial diversity, as a component of wound bioburden, to poor patient outcomes including delayed wound healing [56]. The reduced α-diversity observed in microbiology positive samples may be related to the increased proportion of reads aligned to the pathogen species as the infection progresses. As such, α-diversity could be applied to provide clinicians with additional information on the progress of a wound infection. However, there were not sufficient samples in this study to allow for a comparison of the species diversity according to wound type. Future work should focus on this aspect to increase the understanding of the use of CMg to inform wound management according to microbial species diversity at specific wound types.

There was also a difference observed in the α-diversity from samples collected at each sampling location. This may reflect differences in the environmental contaminants that initially colonise the wound site at each location. Therefore, interpretation of the α-diversity when evaluating a wound infection may need to be location specific. Surprisingly, previous antibiotic treatment did not result in changes to species diversity. It may be expected that antibiotic treatment would reduce species diversity through the removal of sensitive bacteria species, as observed at other sites, such as the gut microbiome [57]. The removal of sensitive species at the wound site by antibiotics may result in increased colonisation by resistant bacteria and fungal species, without impacting the overall species diversity. However, the timeframe of previous antibiotic use was not evaluated in this study and this may also have had an impact on the diversity of the species present at the wound at the time of sampling. Further work should be carried out to understand the relationship between species α-diversity, wound infection severity, antibiotic treatment, including the duration of treatment, and delayed healing.

In addition to identifying the causative pathogen, determining antimicrobial resistance is key to enabling the optimal treatment of a wound infection. These results have demonstrated that AMR genes associated with the resistance observed by AST are identified from CMg sequencing data. However, further understanding of the relationship between the presence of an AMR gene and the phenotypic resistance profile of a pathogen is required. For example, the method development carried out for this study included the evaluation of the detection of the *mecA* gene in *S. aureus*, associated with methicillin resistance. However, the presence of the *mecA* homolog, *mecC*, also confers methicillin resistance in *S. aureus* [58], such that absence of the *mecA* gene cannot guarantee sensitivity to methicillin. In addition, AMR genes were also identified in samples that did not report the presence of a wound pathogen and in samples with colonies determined to be sensitive by AST, potentially originating from the commensal species present in the sample. Further work should be carried out to better understand the consequence of AMR gene identification and identify a comprehensive list of reportable AMR genes associated with specific pathogen species for wound infections.

A key aim of this study was to ensure that the method complexity was minimised to enable the future application of the workflow in a deployed military environment. Adapting methods for use in austere environments could also open up the application of CMg-based diagnostics in environments with limited access to permanent healthcare facilities. However, there was a continued requirement that the sensitivity and specificity of pathogen detection must remain as high as possible to inform patient treatment. Several steps, ordinarily included in CMg workflows that involved large, bulky equipment, were omitted. In addition, specific reagent kits developed for ease-of-use, including the GenomiPhi™ V3 Ready-to-Go™ WGA kit and the nanopore rapid barcoding library preparation kit were included. The use of nanopore-based sequencing itself was employed due to the reduced size of the equipment, robustness against movement and reducing the total turnaround time through enabling real-time data analysis. This technology has previously been demonstrated for point-of-care and in-field diagnostics for a range of sample types [59–61].

Further optimisation of additional steps of the workflow is now required. In a routine clinical context, the use of a spiked control would be a key requirement and future iterations of the workflow should incorporate a spiked control, allowing the identification of inter-run variation and differentiate true negative and false negative results. Method optimisation to reduce the hands-on steps and method complexity via increased method automation will reduce the burden on deployed biomedical scientists. The workflow in this study required approximately 4 h to start sequencing and the samples were sequenced for 16 h to ensure that sufficient reads were generated. The use of a real-time analysis pipeline would further reduce the turnaround time, allowing sequencing runs to continue until a reportable pathogen is identified at the required reporting threshold. With this, it is feasible that the methods developed in this study could be adapted for the rapid diagnosis of wound infections in deployed environments.

## Conclusions

We report the development and evaluation of a CMg workflow for the diagnosis of wound infections. The workflow required approximately 4 h to reach the start of DNA sequencing using methods and equipment suitable for use in austere environments. Whilst the workflow included DNA sequencing for a total of 16 h, it is likely that the total sequencing time could be reduced without impairing the detection of wound pathogens, further reducing the total turnaround time. The results showed good concordance with routine microbiology and qPCR testing and enabled the identification of AMR genes. CMg identified additional reportable wound pathogens including fungal pathogens, demonstrating the advantages of applying untargeted, non-selective diagnostic methods. Whilst this study shows the potential for CMg for deployed diagnostics, increased method automation, understanding of the interpretation of CMg results including a threshold for species identification and a list of reportable AMR genes is required before CMg can be routinely implemented in a deployed healthcare setting.

## Supplementary Information

Supplementary Material 1.

## Data Availability

Sequence data that support the findings of this study have been deposited in the National Center for Biotechnology Information Sequence Read Archive. Sequence data obtained during method development were uploaded to BioProject number PRJNA1147578. Sequence data obtained from the wound swab samples collected from study participants were uploaded to BioProject number PRJNA1146314. The sample accession numbers are listed in Additional File 1 Table 4.

## Abbreviations

AMR: Antimicrobial resistance
API: Analytical profile indexes
AST: Antimicrobial susceptibility testing
bp: Base pair
BNH: Basingstoke and North Hampshire Hospital
CARD: Comprehensive Antibiotic Resistance Database
CFU: Colony forming unit
CMg: Clinical metagenomics
Cq: Quantitation cycle
MALDI-TOF-MS: Matrix-assisted laser desorption/ionisation - time of flight mass spectrometry
MODREC: Ministry of Defence Research Ethics Committee
NCTC: National Collection of Type Cultures
NHS: National Health Service
PCR: Polymerase chain reaction
PBS: phosphate-buffered saline
qPCR: Real-time quantitative polymerase chain reaction
SDH: Salisbury District Hospital
WGA: Whole genome amplification
