# Supplementary material for: Development of a clinical metagenomics workflow for the diagnosis of wound infections
Source: BMC Med Genomics. 2024 Nov 25;17:276. doi: 10.1186/s12920-024-02044-w (PMC11587571; doi:10.1186/s12920-024-02044-w)
Supplement: Supplementary file 1 — Supplementary Material 1. [file 12920_2024_2044_MOESM1_ESM.docx]

**Additional File 1 Table 1.** Wound sample characteristics.

| Sample Number | Wound Type | Wound Location | Antibiotics prescribed prior to sample collection |
| --- | --- | --- | --- |
| 01 | Laceration | Palmar aspect of metacarpophalangeal joint of index finger | No antibiotics at time of sampling. |
| 02 | Animal bite | Leg | No antibiotics at time of sampling |
| 03 | Unspecified non-healing | Ankle | No antibiotics at time of sampling |
| 04 | Animal bite | Finger | No antibiotics at time of sampling |
| 05 | Ulcer | Lateral upper aspect of foot | No antibiotics at time of sampling |
| 06 | Burn | Wound location not specified | Flucloxacillin |
| 07 | Ulcer | Upper aspect of toe | Flucloxacillin |
| 08 | Ulcer | Left medial malleolus | No antibiotics at time of sampling |
| 09 | Ulcer | Lower leg | Flucloxacillin and clindamycin |
| 10 | Ulcer | Lower leg | Flucloxacillin |
| 11 | Ulcer | Hallux | CoAmoxiclav, gentamicin |
| 12 | Non-healing amputation | Toe | No antibiotics at time of sampling |
| 13 | Unspecified non-healing | Plantar aspect of toe | Flucloxacillin |
| 14 | Ulcer | Heel | No antibiotics at time of sampling |
| 15 | Ulcer | Medial aspect of foot over metatarsal head | No antibiotics at time of sampling |
| 16 | Ulcer | Toe apex | No antibiotics at time of sampling |
| 17 | Ulcer | Foot | Clindamycin |
| 18 | Pressure | Sacrum | Clindamycin |
| 19 | Ulcer | Ankle | Amoxicillin, meropenem |
| 20 | Ulcer | Lower leg | Flucloxacillin, gentamycin |
| 21 | Ulcer | Lower leg | Clindamycin |
| 22 | Ulcer | Heel | CoAmoxiclav, flucloxacillin |
| 23 | Ulcer | Lower leg | Flucloxacillin |
| 24 | Ulcer | Heel | Metronidazole, ciprofloxacin, coTrimoxazole |
| 25 | Ulcer | Lower leg | Linezolid, meropenem, clindamycin |
| 26 | Animal bite | Lower leg | Doxycycline, ceftriaxone |
| 27 | Ulcer | Heel | CoAmoxiclav |
| 28 | Ulcer | Foot | No antibiotics at time of sampling |
| 29 | Ulcer | Heel | CoAmoxiclav, flucloxacillin |
| 30 | Ulcer | Lower leg | No antibiotics at time of sampling |
| 31 | Ulcer | Lower leg | No antibiotics at time of sampling |
| 32 | Pressure | Heel | Doxycycline |
| 33 | Ulcer | Ankle | No antibiotics at time of sampling |
| 34 | Ulcer | Lateral foot over metatarsal head | No antibiotics at time of sampling |
| 35 | Unspecified non-healing | Plantar aspect of foot | Antibiotic history unknown |
| 36 | Ulcer | Plantar aspect of foot | Antibiotic history unknown |
| 37 | Ulcer | Lateral aspect of foot | Antibiotic history unknown |
| 38 | Ulcer | Plantar aspect right foot | Antibiotic history unknown |
| 39 | Ulcer | Toe | Co-amoxiclav |
| 40 | Ulcer | Toe | Co-amoxiclav |

Additional wound samples were collected from study participants immediately following the collection of the routine wound swab sample. Wound swab sample characteristics, where available, were provided by the NHS standard of care team.

**Additional File 1 Table 2.** Results of CMg analysis of the wound swab samples.

| Sample Number | Post-depletion DNA Extract Concentration (ng/µL) | Total Reads | Mean Read Length (bp) | Classified Microbial Reads | Human Reads | Organisms identified by metagenomics sequencing (≥ 1% classified microbial reads) | Species reads | % classified microbial reads |
| --- | --- | --- | --- | --- | --- | --- | --- | --- |
| 01 | 2.31 | 217440 | 1505 | 157878 | 32790 | *Enterobacter hormaechei*  *Enterobacter sp. DSM 30060*  *Enterobacter roggenkampii*  *Enterobacter cloacae complex sp. ECL414*  *Klebsiella pneumoniae* | 111837  21481  7014  6398  1707 | 70.84  13.61  4.44  4.05  1.08 |
| 02 | 0.313 | 308679 | 1247 | 8161 | 293523 | *Mycobacteroides chelonae*  *Toxoplasma gondii*  *Mycobacteroides abscessus*  *Mycobacterium stephanolepidis*  *Mycobacteroides salmoniphilum* | 6326  497  318  96  93 | 77.52  6.09  3.90  1.18  1.14 |
| 03 | <0.05 | 196621 | 917 | 2764 | 190054 | *Staphylococcus aureus*  *Toxoplasma gondii*  *Finegoldia magna*  *Lacticaseibacillus paracasei*  *Lentilactobacillus buchneri* | 1555  245  231  35  28 | 64.85  10.22  9.63  1.46  1.17 |
| 04 | <0.05 | 294741 | 927 | 8666 | 171392 | *Pasteurella multocida*  *Tannerella forsythia*  *Fusobacterium nucleatum*  *Porphyromonas gingivalis*  *Toxoplasma gondii*  *Campylobacter sp. CCUG 57310*  *Fusobacterium pseudoperiodonticum*  *Fusobacterium hwasookii*  *Campylobacter sp. RM16192*  *Fusobacterium canifelinum* | 2708  2390  771  480  451  362  170  152  113  105 | 31.25  27.58  8.90  5.54  5.20  4.18  1.96  1.75  1.30  1.21 |
| 05 | 11.7 | 288444 | 1461 | 247600 | 1298 | *Enterococcus faecalis*  *Klebsiella aerogenes* | 232570  8879 | 93.93  3.59 |
| 06 | 0.135 | 39997 | 797 | 112.0 | 38457 | *Staphylococcus epidermis*  *Aspergillus luchuensis*  *Corynebacterium glucuronolyticum* | 60  15  13 | 53.57  13.39  11.61 |
| 07 | 5.94 | 63333 | 1294 | 15474 | 19 | *Streptococcus oralis*  *Parvimonas micra*  *Veillonella parvula*  *Streptococcus sp. oral taxon 064*  *Streptococcus constellatus*  *Streptococcus sp. NSJ-72*  *Streptococcus milleri*  *Prevotella buccalis*  *Anaerococcus obesiensis*  *Anaerococcus vaginalis*  *Streptococcus anginosus*  *Peptoniphilus harei* | 3480  3020  2310  1380  1030  770  716  417  307  234  166  163 | 22.49  19.52  14.93  8.92  6.66  4.98  4.63  2.69  1.98  1.51  1.07  1.05 |
| 08 | 7.88 | 109061 | 1377 | 45571 | 3948 | *Escherichia coli*  *Enterobacter hormaechei*  *Klebsiella pneumoniae*  *Enterococcus faecalis*  *Enterobacter cloacae*  *Corynebacterium jeikeium*  *Citrobacter portucalensis*  *Finegoldia magna*  *Corynebacterium coyleae* | 20441  9940  4420  1840  1810  1780  945  943  457 | 44.77  21.81  9.70  4.04  3.97  3.91  2.07  2.07  1.00 |
| 09 | 0.097 | 5636 | 944 | 642 | 31 | *Rothia dentocariosa*  *Rothia aeria*  *Cutibacterium acnes*  *Corynebacterium tuberculostearicum*  *Staphylococcus epidermidis*  *Staphylococcus haemolyticus*  *Staphylococcus warneri*  *Staphylococcus pasteuri*  *Malassezia restricta* | 405  44  34  33  23  16  12  10  9 | 63.08  6.85  5.30  5.14  3.58  2.49  1.87  1.56  1.40 |
| 10 | 0.170 | 44840 | 1151 | 2735 | 34271 | *Streptococcus pyogenes*  *Staphylococcus aureus* | 1542  1126 | 56.31  41.17 |
| 11 | <0.05 | 2112 | 365 | 51 | 299 | *Dermabacter jinjuensis* | 6 | 11.76 |
| 12 | 0.931 | 64218 | 1461 | 31411.0 | 12773 | *Citrobacter koseri* | 29995 | 95.49 |
| 13 | 7.76 | 34286 | 1194 | 23145 | 48 | *Bacteroides fragilis*  *Streptococcus dysgalactiae*  *Anaerococcus obesiensis*  *Bacteroides ovatus*  *Anaerococcus vaginalis*  *Finegoldia magna*  *Peptoniphilus harei*  *Helcococcus kunzii* | 15096  2830  1010  790  594  561  463  293 | 65.22  12.23  4.36  3.41  2.57  2.42  2.00  1.27 |
| 14 | 4.98 | 43397 | 1084 | 23118 | 7243 | *Staphylococcus aureus* | 23082 | 99.84 |
| 15 | 5.54 | 33311 | 1227 | 15395 | 8541 | *Staphylococcus aureus* | 15365 | 99.81 |
| 16 | 0.156 | 14647 | 876 | 5787 | 2468 | *Staphylococcus aureus*  *Finegoldia magna*  *Corynebacterium striatum*  *Acinetobacter johnsonii* | 1933  1880  1380  138 | 33.40  32.49  23.85  2.38 |
| 17 | 0.559 | 20929 | 963 | 3525 | 856 | *Pseudomonas sp. NIBR-H-19*  *Corynebacterium striatum*  *Klebsiella oxytoca* | 3127  86  68 | 88.71  2.44  1.93 |
| 18 | 0.781 | 151500 | 1474 | 53009 | 303 | *Escherichia coli*  *Lacticaseibacillus rhamnosus*  *Candida albicans*  *Corynebacterium striatum*  *Lactobacillus gasseri*  *Staphylococcus epidermidis* | 35004  8859  3273  2587  1803  537 | 66.03  16.71  6.17  4.88  3.40  1.01 |
| 19 | 0.186 | 43525 | 1212 | 1498 | 32139 | *Corynebacterium striatum*  *Corynebacterium Simulans* | 1396  24 | 93.19  1.60 |
| 20 | 2.77 | 161465 | 1555 | 30104 | 19182 | *Corynebacterium urealyticum*  *Corynebacterium propinquum*  *Corynebacterium aurimucosum*  *Corynebacterium tuberculostearicum*  *Corynebacterium jeikeium* | 16700  7830  1580  469  338 | 55.47  26.01  5.25  1.56  1.12 |
| 21 | 1.08 | 191440 | 1168 | 67266 | 9304 | *Citrobacter koseri*  *Citrobacter sp. TBCP-5362*  *Mammaliicoccus lentus* | 59255  3843  1279 | 88.09  5.71  1.90 |
| 22 | 9.47 | 205000 | 1554 | 59660 | 82 | *Morganella morganii*  *Streptococcus gordonii*  *Anaerococcus obesiensis*  *Finegoldia magna*  *Anaerococcus vaginalis* | 45270  5522  1256  914  899 | 75.88  9.26  2.11  1.53  1.51 |
| 23 | 1.22 | 150218 | 1433 | 38028 | 22082 | *Corynebacterium striatum* | 36283 | 95.41 |
| 24 | 1.71 | 43689 | 1347 | 8437 | 1350 | *Candida albicans*  *Enterococcus faecalis*  *Corynebacterium striatum*  *Staphylococcus haemolyticus* | 3288  2495  2259  217 | 38.97  29.57  26.77  2.57 |
| 25 | 0.989 | 70854 | 1309 | 18975 | 581 | *Staphylococcus pseudintermedius* | 17986 | 94.79 |
| 26 | <0.05 | 43218 | 988 | 8430 | 6111 | *Streptococcus pyogenes*  *Staphylococcus aureus*  *Staphylococcus epidermidis* | 6994  544  538 | 82.97  6.45  6.38 |
| 27 | 0.337 | 75430 | 1172 | 10952 | 14475 | *Corynebacterium striatum*  *Finegoldia magna*  *Anaerococcus vaginalis*  *Anaerococcus obesiensis*  *Staphylococcus aureus*  *Corynebacterium simulans* | 5646  1156  1140  1100  868  130 | 51.55  10.56  10.41  10.04  7.93  1.19 |
| 28 | 1.83 | 395134 | 1737 | 42295 | 13010 | *Corynebacterium striatum*  *Rhodococcus erythropolis*  *Finegoldia magna*  *Rhodococcus sp. P-2*  *Pseudomonas aeruginosa*  *Anaerococcus sp. Marseille-Q7828*  *Peptoniphilus harei*  *Anaerococcus mediterraneensis*  *Schaalia radingae*  *Helcococcus kunzii* | 30433  1541  1356  1334  1294  1173  1006  943  607  592 | 71.95  3.64  3.21  3.15  3.06  2.77  2.38  2.23  1.44  1.40 |
| 29 | 0.368 | 34373 | 779 | 95 | 31336 | *Staphylococcus aureus*  *Toxoplasma gondii* | 62  21 | 65.26  22.11 |
| 30 | 21.6 | 292064 | 2046 | 122166 | 23705 | *Pseudomonas aeruginosa*  *Pseudomonas putida* | 101154  18103 | 82.80  14.82 |
| 31 | <0.05 | 1993 | 651 | 218 | 756 | *Staphylococcus simulans*  *Staphylococcus epidermidis* | 175  15 | 80.28  6.88 |
| 32 | 0.091 | 42928 | 1220 | 16523 | 2544 | *Corynebacterium striatum*  *Streptococcus dysgalactiae*  *Dermabacter jinjuensis*  *Finegoldia magna*  *Enterococcus faecalis*  *Corynebacterium simulans* | 9621  4783  424  320  217  184 | 58.23  28.95  2.57  1.94  1.31  1.11 |
| 33 | 10.9 | 200437 | 1765 | 95559 | 433 | *Bacteroides fragilis*  *Morganella morganii*  *Peptoniphilus sp. SAHP1*  *Corynebacterium striatum*  *Bacteroides uniformis*  *Proteus mirabilis* | 70466  10540  3211  2297  2221  1938 | 73.74  11.03  3.36  2.40  2.32  2.03 |
| 34 | 1.19 | 22931 | 1474 | 13068 | 2269 | *Corynebacterium striatum*  *Pseudomonas aeruginosa*  *Corynebacterium simulans* | 10922  1224  153 | 83.58  9.37  1.17 |
| 35 | 1.27 | 40228 | 1346 | 18067 | 85 | *Staphylococcus pettenkoferi*  *Corynebacterium striatum*  *Streptococcus dysgalactiae*  *Enterococcus faecalis*  *Staphylococcus simulans*  *Helcococcus kunzii*  *Staphylococcus aureus*  *Staphylococcus epidermidis* | 9809  3799  1239  1198  510  260  252  182 | 54.29  21.03  6.86  6.63  2.82  1.44  1.39  1.01 |
| 36 | 20.2 | 38948 | 1285 | 23440 | 393.00 | *Streptococcus dysgalactiae*  *Staphylococcus aureus*  *Corynebacterium striatum*  *Streptococcus pyogenes*  *Streptococcus agalactiae*  *Pseudomonas aeruginosa* | 18000  1377  1267  631  547  314 | 76.79  5.87  5.41  2.69  2.33  1.34 |
| 37 | 7.23 | 44806 | 1119 | 27416 | 27 | *Streptococcus dysgalactiae*  *Prevotella corporis*  *Bacteroides fragilis*  *Streptococcus pyogenes*  *Streptococcus agalactiae*  *Peptoniphilus sp. SAHP1*  *Finegoldia magna* | 20847  2089  1180  722  452  312  278 | 76.04  7.62  4.30  2.63  1.65  1.14  1.01 |
| 38 | 5.66 | 56748 | 1594 | 32422 | 13.00 | *Klebsiella michiganensis*  *Streptococcus anginosus*  *Klebsiella oxytoca*  *Corynebacterium striatum*  *Klebsiella pneumoniae*  *Citrobacter freundii*  *Enterococcus faecalis*  *Anaerococcus obesiensis*  *Bacteroides fragilis*  *Klebsiella grimontii*  *Peptoniphilus sp. SAHP1*  *Parvimonas micra*  *Anaerococcus vaginalis*  *Escherichia coli*  *Pseudomonas aeruginosa*  *Enterobacter hormaechei*  *Morganella morganii* | 12535  3261  1667  1475  1390  1269  1182  890  757  731  713  658  617  576  463  442  329 | 38.66  10.06  5.14  4.55  4.29  3.91  3.65  2.75  2.33  2.25  2.20  2.03  1.90  1.78  1.43  1.36  1.01 |
| 39 | <0.05 | 36541 | 1253 | 7929 | 6088 | *Finegoldia magna*  *Staphylococcus simulans*  *Anaerococcus obesiensis*  *Anaerococcus vaginalis*  *Peptoniphilus harei*  *Helcococcus kunzii*  *Anaerococcus mediterraneensis*  *Staphylococcus pettenkoferi*  *Streptococcus pyogenes*  *Streptococcus agalactiae* | 2946  830  625  589  359  347  313  129  103  97 | 37.15  10.47  7.88  7.43  4.53  4.38  3.95  1.63  1.30  1.22 |
| 40 | 3.52 | 102369 | 1626 | 22819 | 1986 | *Alcaligenes faecalis*  *Finegoldia magna*  *Helcococcus kunzii*  *Anaerococcus sp. Marseille-Q7828*  *Corynebacterium aurimucosum*  *Anaerococcus obesiensis*  *Peptoniphilus harei*  *Corynebacterium amycolatum*  *Anaerococcus vaginalis*  *Anaerococcus mediterraneensis*  *Pseudomonas aeruginosa*  *Corynebacterium jeikeium*  *Anaerococcus prevotii*  *Corynebacterium sp. ATCC 6931*  *Fastidiosipila sanguinis* | 4218  4110  2203  983  903  787  747  737  664  587  518  408  395  347  233 | 18.48  18.01  9.65  4.31  3.96  3.45  3.27  3.23  2.91  2.57  2.27  1.79  1.73  1.52  1.02 |

Additional wound swabs collected from study participants were processed for CMg. DNA extracts were quantified prior to WGA and library preparation using the Qubit dsDNA HS assay. Human reads were quantified by aligning sequences to a reference human genome and then removed prior to further analysis. All microbial species identified by sequencing ≥1% of classified microbial reads are reported. % classified microbial reads = total number of reads assigned to a species over the total number of taxonomically classified microbial reads obtained × 100.

**Additional File 1 Table 3.** Detection of high priority wound pathogen species by microbiology testing, qPCR and CMg.

| Sample Number | *E.coli* | | | | *P. aeruginosa* | | | | *S. aureus* | | | | | | | *S. pyogenes* | | | | | |
| --- | --- | --- | --- | --- | --- | --- | --- | --- | --- | --- | --- | --- | --- | --- | --- | --- | --- | --- | --- | --- | --- |
|  | Species reported by microbiology testing (Yes/No) | qPCR Cq | CMg Reads (% classified microbial reads) | Average read length (bp) | Species reported by microbiology testing (Yes/No) | qPCR Cq | CMg Reads (% classified microbial reads) | Average read length (bp) | Species reported by microbiology testing (Yes/No) | | qPCR Cq | | CMg Reads (% classified microbial reads) | | Average read length (bp) | Species reported by microbiology testing (Yes/No) | | qPCR Cq | CMg Reads (% classified microbial reads) | | Average read length (bp) |
| 01 | No | No detection | 935 (0.59) | 900.04 | No | No detection | 6 (0.01) | 1823.17 | No detection | | | | | | | No detection | | | | | |
| 02 | No detection | | | | No detection | | | | No | | No detection | | 26 (0.32) | | 1189.15 | No detection | | | | | |
| 03 | No detection | | | | No detection | | | | Yes | | 34.29 | | 1555 (64.85) | | 793.02 | No detection | | | | | |
| 04 | No detection | | | | No detection | | | | No | | No detection | | 32 (0.37) | | 636.36 | No detection | | | | | |
| 05 | No | 25.13 | 251 (0.10) | 1536.50 | No | 34.45 | 0 (0.00) | - | No | | 34.15 | | 854 (0.34) | | 1072.55 | No detection | | | | | |
| 06 | No detection | | | | No detection | | | | No detection | | | | | | | No detection | | | | | |
| 07 | No detection | | | | No detection | | | | No | No detection | | 6 (0.04) | | 793.66 | | No | | No detection | 39 (0.25) | | 1342.90 |
| 08 | Yes | 19.29 | 20441 (44.77) | 1391.72 | No detection | | | | No detection | | | | | | | No | | No detection | 8 (0.02) | | 1163.14 |
| 09 | No detection | | | | No detection | | | | No detection | | | | | | | No detection | | | | | |
| 10 | No detection | | | | No detection | | | | Yes | | 24.91 | | 1126 (41.17) | | 998.23 | No | | 26.47 | 1542 (56.31) | | 1074.60 |
| 11 | No detection | | | | No detection | | | | Yes | | 32.47 | | 0 (0.00) | | - | No detection | | | | | |
| 12 | No | No detection | 120 (0.38) | 594.44 | No detection | | | | No detection | | | | | | | No detection | | | | | |
| 13 | No detection | | | | No detection | | | | No | | 36.18 | | 0 (0.00) | | - | No | | No detection | 138 (0.60) | | 682.46 |
| 14 | No detection | | | | No detection | | | | Yes | | 18.01 | | 23082 (99.84) | | 878.67 | No detection | | | | | |
| 15 | No detection | | | | No detection | | | | Yes | | 19.13 | | 15365 (99.81) | | 911.72 | No detection | | | | | |
| 16 | No detection | | | | No detection | | | | Yes | | 25.78 | | 1933 (33.40) | | 745.26 | No | | No detection | 25 (0.43) | | 800.71 |
| 17 | No | No detection | 20 (0.57) | 1873.63 | No detection | | | | No detection | | | | | | | No detection | | | | | |
| 18 | Yes | 21.02 | 35004 (66.03) | 1366.20 | No detection | | | | No | | No detection | | 29 (0.06) | | 1336.12 | No detection | | | | | |
| 19 | No detection | | | | No detection | | | | No detection | | | | | | | No detection | | | | | |
| 20 | No | No detection | 23 (0.08) | 976.61 | No | No detection | 27 (0.09) | 1342.40 | Yes | | 27.51 | | 186 (0.62) | | 1074.10 | No detection | | | | | |
| 21 | No | No detection | 36 (0.05) | 735.15 | No detection | | | | Yes | | 35.02 | | 94 (0.14) | | 950.76 | No | | No detection | 9 (0.01) | | 1533.50 |
| 22 | No | No detection | 72 (0.12) | 1515.62 | No | No detection | 26 (0.04) | 1408.39 | No detection | | | | | | | No | | No detection | 29 (0.05) | | 1294.08 |
| 23 | No detection | | | | No detection | | | | Yes | | 28.85 | | 212 (0.56) | | 907.75 | No | No detection | | 10 (0.03) | 831.67 | |
| 24 | No detection | | | | No detection | | | | No | | No detection | | 42 (0.50) | | 1034.74 | No | | No detection | 6 (0.07) | | 775.50 |
| 25 | No detection | | | | No detection | | | | No | | No detection | | 32 (0.17) | | 1019.96 | No | | 24.14 | 125 (0.66) | | 1052.53 |
| 26 | No detection | | | | No detection | | | | Yes | | 31.53 | | 544 (6.45) | | 964.70 | Yes | | 29.13 | 6994 (82.97) | | 1019.32 |
| 27 | No | 36.06 | 0 (0.00) | - | No detection | | | | Yes | | 27.67 | | 868 (7.93) | | 921.96 | No | | No detection | 28 (0.26) | | 966.19 |
| 28 | No detection | | | | Yes | 26.93 | 1294 (3.06) | 1508.12 | No | | No detection | | 10 (0.02) | | 996.80 | No | | No detection | 56 (0.13) | | 1151.87 |
| 29 | No detection | | | | No detection | | | | Yes | | 28.18 | | 62 (65.26) | | 1014.03 | No detection | | | | | |
| 30 | No detection | | | | No | 17.70 | 101154 (82.80) | 1742.53 | No detection | | | | | | | No detection | | | | | |
| 31 | No detection | | | | No detection | | | | No detection | | | | | | | No detection | | | | | |
| 32 | No detection | | | | No detection | | | | No | | No detection | | 5 (0.03) | | 1092.00 | No | | No detection | 54 (0.32) | | 785.56 |
| 33 | No | 26.93 | 39 (0.04) | 1544.59 | No | No detection | 536 (0.56) | 2493.68 | No | | 34.76 | | 11 (0.01) | | 1223.44 | No | | No detection | 54 (0.06) | | 1227.55 |
| 34 | No detection | | | | Yes | 26.48 | 1224 (9.37) | 1061.42 | No detection | | | | | | | No detection | | | | | |
| 35 | No | 37.81 | 0 (0.00) | - | No | 30.25 | 20 (0.11) | 1234.06 | Yes | | 34.82 | | 252 (1.39) | | 575.16 | No | | No detection | 48 (0.27) | | 549.17 |
| 36 | No | 26.48 | 43 (0.18) | 1030.63 | No | 22.44 | 314 (1.34) | 1245.78 | Yes | | 22.46 | | 1377 (5.87) | | 728.68 | No | | No detection | 631 (2.69) | | 442.17 |
| 37 | No detection | | | | No | 34.57 | 0 (0.00) | - | Yes | | 29.39 | | 23 (0.08) | | 691.43 | No | | No detection | 722 (2.63) | | 454.00 |
| 38 | No | No detection | 576 (1.78) | 1372.05 | No | 22.35 | 463 (1.43) | 1199.77 | No | | No detection | | 9 (0.03) | | 491.22 | No | | No detection | 14 (0.04) | | 536.91 |
| 39 | No detection | | | | No detection | | | | No | | 37.53 | | 75 (0.95) | | 598.06 | No | | No detection | 103 (1.30) | | 674.48 |
| 40 | No | 33.22 | 0 (0.00) | - | No | 23.84 | 518 (2.27) | 1150.95 | No | | 34.67 | | 85 (0.37) | | 1013.27 | No | | No detection | 145 (0.64) | | 652.39 |

The DNA extract from each additional wound swab sample was tested for four high priority pathogen species by qPCR. The routine microbiology testing report, qPCR Cq value and the number of reads assigned to the target species by CMg are reported. % classified microbial reads = % of species reads over the total number of taxonomically classified microbial reads obtained.

**Additional File 1 Table 4.** Sequence data availability.

| Sample Number | BioProject Number | Sample description | Sequencing Run | Sample Accession |
| --- | --- | --- | --- | --- |
| 01 | PRJNA1146314 | Wound swab sample collected from study participant. | 1 | SAMN43089555 |
| 02 | PRJNA1146314 | Wound swab sample collected from study participant. | 1 | SAMN43089556 |
| 03 | PRJNA1146314 | Wound swab sample collected from study participant. | 1 | SAMN43089557 |
| 04 | PRJNA1146314 | Wound swab sample collected from study participant. | 1 | SAMN43089558 |
| 05 | PRJNA1146314 | Wound swab sample collected from study participant. | 1 | SAMN43089559 |
| 06 | PRJNA1146314 | Wound swab sample collected from study participant. | 2 | SAMN43089560 |
| 07 | PRJNA1146314 | Wound swab sample collected from study participant. | 2 | SAMN43089561 |
| 08 | PRJNA1146314 | Wound swab sample collected from study participant. | 2 | SAMN43089562 |
| 09 | PRJNA1146314 | Wound swab sample collected from study participant. | 2 | SAMN43089563 |
| 10 | PRJNA1146314 | Wound swab sample collected from study participant. | 2 | SAMN43089564 |
| 11 | PRJNA1146314 | Wound swab sample collected from study participant. | 3 | SAMN43089565 |
| 12 | PRJNA1146314 | Wound swab sample collected from study participant. | 3 | SAMN43089566 |
| 13 | PRJNA1146314 | Wound swab sample collected from study participant. | 3 | SAMN43089567 |
| 14 | PRJNA1146314 | Wound swab sample collected from study participant. | 3 | SAMN43089568 |
| 15 | PRJNA1146314 | Wound swab sample collected from study participant. | 3 | SAMN43089569 |
| 16 | PRJNA1146314 | Wound swab sample collected from study participant. | 4 | SAMN43089570 |
| 17 | PRJNA1146314 | Wound swab sample collected from study participant. | 4 | SAMN43089571 |
| 18 | PRJNA1146314 | Wound swab sample collected from study participant. | 4 | SAMN43089572 |
| 19 | PRJNA1146314 | Wound swab sample collected from study participant. | 4 | SAMN43089573 |
| 20 | PRJNA1146314 | Wound swab sample collected from study participant. | 4 | SAMN43089574 |
| 21 | PRJNA1146314 | Wound swab sample collected from study participant. | 4 | SAMN43089575 |
| 22 | PRJNA1146314 | Wound swab sample collected from study participant. | 4 | SAMN43089576 |
| 23 | PRJNA1146314 | Wound swab sample collected from study participant. | 5 | SAMN43089577 |
| 24 | PRJNA1146314 | Wound swab sample collected from study participant. | 5 | SAMN43089578 |
| 25 | PRJNA1146314 | Wound swab sample collected from study participant. | 5 | SAMN43089579 |
| 26 | PRJNA1146314 | Wound swab sample collected from study participant. | 5 | SAMN43089580 |
| 27 | PRJNA1146314 | Wound swab sample collected from study participant. | 5 | SAMN43089581 |
| 28 | PRJNA1146314 | Wound swab sample collected from study participant. | 6 | SAMN43089582 |
| 29 | PRJNA1146314 | Wound swab sample collected from study participant. | 6 | SAMN43089583 |
| 30 | PRJNA1146314 | Wound swab sample collected from study participant. | 6 | SAMN43089584 |
| 31 | PRJNA1146314 | Wound swab sample collected from study participant. | 6 | SAMN43089585 |
| 32 | PRJNA1146314 | Wound swab sample collected from study participant. | 6 | SAMN43089586 |
| 33 | PRJNA1146314 | Wound swab sample collected from study participant. | 6 | SAMN43089587 |
| 34 | PRJNA1146314 | Wound swab sample collected from study participant. | 7 | SAMN43089588 |
| 35 | PRJNA1146314 | Wound swab sample collected from study participant. | 7 | SAMN43089589 |
| 36 | PRJNA1146314 | Wound swab sample collected from study participant. | 7 | SAMN43089590 |
| 37 | PRJNA1146314 | Wound swab sample collected from study participant. | 7 | SAMN43089591 |
| 38 | PRJNA1146314 | Wound swab sample collected from study participant. | 7 | SAMN43089592 |
| 39 | PRJNA1146314 | Wound swab sample collected from study participant. | 8 | SAMN43089593 |
| 40 | PRJNA1146314 | Wound swab sample collected from study participant. | 8 | SAMN43089594 |
| Run 1 Negative Control | PRJNA1146314 | Blank wound swab negative control. | 1 | SAMN43089595 |
| Run 2 Negative Control | PRJNA1146314 | Blank wound swab negative control. | 2 | SAMN43089596 |
| Run 3 Negative Control | PRJNA1146314 | Blank wound swab negative control. | 3 | SAMN43089597 |
| Run 4 Negative Control | PRJNA1146314 | Blank wound swab negative control. | 4 | SAMN43089598 |
| Run 5 Negative Control | PRJNA1146314 | Blank wound swab negative control. | 5 | SAMN43089599 |
| Run 6 Negative Control | PRJNA1146314 | Blank wound swab negative control. | 6 | SAMN43089600 |
| Run 7 Negative Control | PRJNA1146314 | Blank wound swab negative control. | 7 | SAMN43089601 |
| Run 8 Negative Control | PRJNA1146314 | Blank wound swab negative control. | 8 | No reads |
| Negative control Run 1 | PRJNA1147578 | Contrived wound swab sample negative control | Method development Run 1 | SAMN43156728 |
| E. coli Host-depleted Rep 1 | PRJNA1147578 | Contrived wound swab sample spiked with *E. coli*  and host depleted. | Method development Run 1 | SAMN43156729 |
| E. coli Host-depleted Rep 2 | PRJNA1147578 | Contrived wound swab sample spiked with *E. coli*  and host depleted. | Method development Run 1 | SAMN43156730 |
| E. coli Host-depleted Rep 3 | PRJNA1147578 | Contrived wound swab sample spiked with *E. coli*  and host depleted. | Method development Run 1 | SAMN43156731 |
| E. coli Non-depleted Rep 1 | PRJNA1147578 | Contrived wound swab sample spiked with *E. coli.* No host depletion. | Method development Run 1 | SAMN43156732 |
| E. coli Non-depleted Rep 2 | PRJNA1147578 | Contrived wound swab sample spiked with *E. coli.* No host depletion. | Method development Run 1 | SAMN43156733 |
| E. coli Non-depleted Rep 3 | PRJNA1147578 | Contrived wound swab sample spiked with *E. coli.* No host depletion. | Method development Run 1 | SAMN43156734 |
| Negative control 2 Rep 1 | PRJNA1147578 | Contrived wound swab sample negative control | Method development Run 2 | SAMN43156735 |
| Negative control 2 Rep 2 | PRJNA1147578 | Contrived wound swab sample negative control | Method development Run 2 | SAMN43156736 |
| Negative control 2 Rep 3 | PRJNA1147578 | Contrived wound swab sample negative control | Method development Run 2 | SAMN43156737 |
| E. coli LoD 1 Rep 1 | PRJNA1147578 | Contrived wound swab sample spiked with 0.5 × 10^4^ CFU *E. coli* and host depleted. | Method development Run 2 | SAMN43156738 |
| E. coli LoD 1 Rep 2 | PRJNA1147578 | Contrived wound swab sample spiked with 0.5 × 10^4^ CFU *E. coli* and host depleted. | Method development Run 2 | SAMN43156739 |
| E. coli LoD 1 Rep 3 | PRJNA1147578 | Contrived wound swab sample spiked with 0.5 × 10^4^ CFU *E. coli* and host depleted. | Method development Run 2 | SAMN43156740 |
| E. coli LoD 2 Rep 1 | PRJNA1147578 | Contrived wound swab sample spiked with 0.5 × 10^3^ CFU *E. coli* and host depleted. | Method development Run 2 | SAMN43156741 |
| E. coli LoD 2 Rep 2 | PRJNA1147578 | Contrived wound swab sample spiked with 0.5 × 10^3^ CFU *E. coli* and host depleted. | Method development Run 2 | SAMN43156742 |
| E. coli LoD 2 Rep 3 | PRJNA1147578 | Contrived wound swab sample spiked with 0.5 × 10^3^ CFU *E. coli* and host depleted. | Method development Run 2 | SAMN43156743 |
| E. coli LoD 3 Rep 1 | PRJNA1147578 | Contrived wound swab sample spiked with 0.5 × 10^2^ CFU *E. coli* and host depleted. | Method development Run 2 | SAMN43156744 |
| E. coli LoD 3 Rep 2 | PRJNA1147578 | Contrived wound swab sample spiked with 0.5 × 10^2^ CFU *E. coli* and host depleted. | Method development Run 2 | SAMN43156745 |
| E. coli LoD 3 Rep 3 | PRJNA1147578 | Contrived wound swab sample spiked with 0.5 × 10^2^ CFU *E. coli* and host depleted. | Method development Run 2 | SAMN43156746 |
| Negative control 3 | PRJNA1147578 | Contrived wound swab sample negative control | Method development Run 3 | SAMN43156747 |
| S. aureus Host-depleted Rep 1 | PRJNA1147578 | Contrived wound swab sample spiked with *S. aureus* and host depleted. | Method development Run 3 | SAMN43156748 |
| S. aureus Host-depleted Rep 2 | PRJNA1147578 | Contrived wound swab sample spiked with *S. aureus* and host depleted. | Method development Run 3 | SAMN43156749 |
| S. aureus Host-depleted Rep 3 | PRJNA1147578 | Contrived wound swab sample spiked with *S. aureus* and host depleted. | Method development Run 3 | SAMN43156750 |
| S. aureus Non-depleted Rep 1 | PRJNA1147578 | Contrived wound swab sample spiked with *S. aureus*. No host depletion. | Method development Run 3 | SAMN43156751 |
| S. aureus Non-depleted Rep 2 | PRJNA1147578 | Contrived wound swab sample spiked with *S. aureus*. No host depletion. | Method development Run 3 | SAMN43156752 |
| S. aureus Non-depleted Rep 3 | PRJNA1147578 | Contrived wound swab sample spiked with *S. aureus*. No host depletion. | Method development Run 3 | SAMN43156753 |
| Negative control 4 Rep 1 | PRJNA1147578 | Contrived wound swab sample negative control | Method development Run 4 | SAMN43156754 |
| Negative control 4 Rep 2 | PRJNA1147578 | Contrived wound swab sample negative control | Method development Run 4 | SAMN43156755 |
| Negative control 4 Rep 3 | PRJNA1147578 | Contrived wound swab sample negative control | Method development Run 4 | SAMN43156756 |
| S. aureus LoD 1 Rep 1 | PRJNA1147578 | Contrived wound swab sample spiked with 0.5 × 10^4^ CFU *S. aureus* and host depleted. | Method development Run 4 | SAMN43156757 |
| S. aureus LoD 1 Rep 2 | PRJNA1147578 | Contrived wound swab sample spiked with 0.5 × 10^4^ CFU *S. aureus* and host depleted. | Method development Run 4 | SAMN43156758 |
| S. aureus LoD 1 Rep 3 | PRJNA1147578 | Contrived wound swab sample spiked with 0.5 × 10^4^ CFU *S. aureus* and host depleted. | Method development Run 4 | SAMN43156759 |
| S. aureus LoD 2 Rep 1 | PRJNA1147578 | Contrived wound swab sample spiked with 0.5 × 10^3^ CFU *S. aureus* and host depleted. | Method development Run 4 | SAMN43156760 |
| S. aureus LoD 2 Rep 2 | PRJNA1147578 | Contrived wound swab sample spiked with 0.5 × 10^3^ CFU *S. aureus* and host depleted. | Method development Run 4 | SAMN43156761 |
| S. aureus LoD 2 Rep 3 | PRJNA1147578 | Contrived wound swab sample spiked with 0.5 × 10^3^ CFU *S. aureus* and host depleted. | Method development Run 4 | SAMN43156762 |
| S. aureus LoD 3 Rep 1 | PRJNA1147578 | Contrived wound swab sample spiked with 0.5 × 10^2^ CFU *S. aureus* and host depleted. | Method development Run 4 | SAMN43156763 |
| S. aureus LoD 3 Rep 2 | PRJNA1147578 | Contrived wound swab sample spiked with 0.5 × 10^2^ CFU *S. aureus* and host depleted. | Method development Run 4 | SAMN43156764 |
| S. aureus LoD 3 Rep 3 | PRJNA1147578 | Contrived wound swab sample spiked with 0.5 × 10^2^ CFU *S. aureus* and host depleted. | Method development Run 4 | SAMN43156765 |

Sequence data for each sample that contributed to the conclusions of the study were uploaded to the Sequence Read Archive under the project numbers and sample accession numbers described in the table.
